# Femtosecond Laser-Assisted Cataract Surgery Versus Phacoemulsification Cataract Surgery (FACT): A Randomized Noninferiority Trial

**DOI:** 10.1016/j.ophtha.2020.02.028

**Published:** 2020-08

**Authors:** Alexander C. Day, Jennifer M. Burr, Kate Bennett, Catey Bunce, Caroline J. Doré, Gary S. Rubin, Mayank A. Nanavaty, Kamaljit S. Balaggan, Mark R. Wilkins, FACT group, Francesco Aiello, Muna Ali, Bruce Allan, Hayley Boston, Torsten Chandler, Sandeep Dhallu, Ahmed Elkarmouty, Joanna Gambell, Rachael Hunter, Felicia Ikeji, Balasubramaniam Ilango, Emma Jones, Gemma Jones, John Koshy, Nicola Lau, Vincenzo Maurino, Kirithika Muthusamy, Jeffrey Round, Jasmin Singh, Yvonne Sylvestre, Richard Wormald, Yit Yang

**Affiliations:** 1 The NIHR Biomedical Research Centre at Moorfields Eye Hospital NHS Foundation Trust, London, United Kingdom; 2 Moorfields Eye Hospital, London, United Kingdom; 3 UCL Institute of Ophthalmology, London, United Kingdom; 4 School of Medicine, University of St. Andrews, Fife, Scotland; 5 UCL Comprehensive Clinical Trials Unit, London, United Kingdom; 6 Department of Primary Care & Public Health Sciences, King’s College London, London, United Kingdom; 7 Sussex Eye Hospital, Brighton & Sussex University Hospitals NHS Trust, Eastern Road, Brighton, United Kingdom; 8 Wolverhampton and Midlands Eye Infirmary, New Cross Hospital, Royal Wolverhampton NHS Trust, Wolverhampton, West Midlands, United Kingdom

**Keywords:** CI, confidence interval, D, diopters, FACT, Femtosecond Laser-Assisted Cataract Trial, FLACS, femtosecond laser-assisted cataract surgery, IOL, intraocular lens, logMAR, logarithm of the minimum angle of resolution, NHS, National Health Service, PCS, phacoemulsification cataract surgery, RCT, randomized controlled trial, UDVA, unaided distance visual acuity

## Abstract

**Purpose:**

To report the 3-month results of a randomized trial (Femtosecond Laser-Assisted Cataract Trial [FACT]) comparing femtosecond laser-assisted cataract surgery (FLACS) with standard phacoemulsification cataract surgery (PCS).

**Design:**

Multicenter, randomized controlled trial funded by the UK National Institute of Health Research (HTA 13/04/46/).

**Participants:**

Seven hundred eighty-five patients with age-related cataract.

**Methods:**

This trial took place in 3 hospitals in the UK National Health Service (NHS). Randomization (1:1) was stratified by site, surgeon, and 1 or both eyes eligible using a secure web-based system. Postoperative assessments were masked to the allocated intervention. The primary outcome was unaided distance visual acuity (UDVA) in the study eye at 3 months. Secondary outcomes included corrected distance visual acuity, complications, and patient-reported outcomes measures. The noninferiority margin was 0.1 logarithm of the minimum angle of resolution (logMAR). ISRCTN.com registry, number ISRCTN77602616.

**Main Outcome Measures:**

We enrolled 785 participants between May 2015 and September 2017 and randomly assigned 392 to FLACS and 393 to PCS. At 3 months postoperatively, mean UDVA difference between treatment arms was −0.01 logMAR (−0.05 to 0.03), and mean corrected distance visual acuity difference was −0.01 logMAR (95% confidence interval [CI], −0.05 to 0.02). Seventy-one percent of both FLACS and PCS cases were within ±0.5 diopters (D) of the refractive target, and 93% of FLACS and 92% of PCS cases were within ±1.0 D. There were 2 posterior capsule tears in the PCS arm and none in the FLACS arm. There were no significant differences between arms for any secondary outcome.

**Conclusions:**

Femtosecond laser-assisted cataract surgery is not inferior to conventional PCS surgery 3 months after surgery. Both methods are as good in terms of vision, patient-reported health, and safety outcomes at 3 months. Longer-term outcomes of the clinical effectiveness and cost-effectiveness are awaited.

Cataract surgery is one of the most commonly performed operations in the western world, with approximately half a million performed per year in the United Kingdom^1^ alone. The current standard method, phacoemulsification (ultrasound) cataract surgery (PCS), was introduced more than 50 years ago.^2^ Femtosecond laser-assisted cataract surgery (FLACS) first became commercially available approximately 10 years ago. Reported advantages include more accurate positioning, shape, and size of the capsulotomy when compared with a capsulorhexis^3^, ^4^, ^5^ and less intraocular lens (IOL) tilt^6^ with fewer higher-order aberrations.^7^ Also, by using a laser to fragment the crystalline lens, less ultrasound energy is subsequently required to complete its removal,^4^ and there is lower endothelial cell loss.^4^^,^^5^ Overall, this would be expected to translate to greater safety and better visual outcomes through greater precision and reproducibility.

At introduction, laser cataract surgery platforms were marketed as bringing a stepwise improvement in surgical technique and were used as a differentiating factor between cataract surgery providers. The cost of FLACS still remains high, reflecting the development costs with, for example, Alcon (Fort Worth, TX) taking over LenSx for $744 million in 2010^8^ and Abbott Medical Optics (Santa Ana, CA) purchasing Optimedica for up to $400 million in 2013.^9^ In an economic modeling evaluation on a simulated cohort of patients undergoing FLACS compared with conventional PCS, FLACS was not cost-effective.^10^ This finding was based on a hypothetical cohort, and robust data from randomized controlled trials (RCTs) are needed to investigate FLACS versus PCS. To date, there are limited high-quality data from RCTs on outcomes for FLACS compared with PCS, with that available being predominantly from large comparative case series.^11^, ^12^, ^13^ The 2016 Cochrane Review of FLACS versus PCS concluded there was limited evidence to determine equivalence or superiority and that large adequately powered RCTs were needed.^11^ Three meta-analyses have been published,^12^, ^13^, ^14^ one finding superior refractive outcomes for FLACS, and the others finding no statistically significant differences in terms of patient-reported visual and refractive complications. Two large RCTs have recently been published: the French FEMCAT (Femtosecond Laser Assisted Cataract Surgery) trial,^15^ and a UK trial of 400 eyes that found similar visual outcomes between arms and a statistically significantly lower posterior capsule tear rate in the laser arm.^16^

The aim of this trial, Femtosecond Laser-Assisted Cataract Trial (FACT), is to establish whether FLACS is as good as or better than conventional PCS.^17^

## Methods

### Design and Patients

The FACT trial was a pragmatic, multicenter, single-masked, randomized controlled trial performed at 3 hospitals in the United Kingdom to compare FLACS with PCS (ISRCTN.com registry number ISRCTN77602616).^17^ The 3 trial sites were high-volume National Health Service (NHS) daycare surgery units (Moorfields at St. Ann’s Hospital, Tottenham, UK; Sussex Eye Hospital, Brighton, UK; and New Cross Hospital, Wolverhampton, UK). The trial received ethical approval by the National Research Ethics Service (NRES) Committee London, City Road & Hampstead (06/02/2015, ref: 14/LO/1937). The design of the trial is detailed in full in the published protocol,^17^^,^^18^ and the final version v4.0 is available online (https://fundingawards.nihr.ac.uk/award/13/04/46). The trial adhered to the tenets of the Declaration of Helsinki.

All patients were screened and recruited from routine cataract clinics between May 2015 and September 2017. Adults aged 18 years or older with age-related cataract with expected postoperative refractive target within ±0.5 diopters (D) of emmetropia (i.e., good distance vision) were eligible for participation. Full inclusion and exclusion criteria are provided in the Appendix (available at www.aaojournal.org). All patients provided written informed consent before participation.

### Randomization and Masking

Participants were randomly assigned in a 1:1 ratio to undergo FLACS or conventional PCS. Randomization was performed on the day of surgery using a web-based, online, sealed envelope-based system (https://www.sealedenvelope.com) that used treatment center, surgeon, and 1 or both eyes eligible as minimization stratifiers. For participants who required bilateral cataract surgery, the same intervention (i.e., FLACS or PCS) was offered when the patient returned for second eye surgery unless the patient stated otherwise. Where possible, the second eye received operation within 8 weeks of the first. Because of the nature of the intervention, surgeon and participant masking were not possible. All trial follow-ups were performed by an optometrist masked to the trial intervention.

### Procedures

All participants underwent dilated slit-lamp examination before listing for cataract surgery by an ophthalmologist. Patients with 1 or both eyes eligible were treated identically. All participants had conventional PCS or FLACS with the Catalys femtosecond laser (Johnson & Johnson Inc., New Brunswick, NJ; St. Ann’s Moorfields Eye Hospital, London, UK) or Ziemer LDV 8 (Ziemer Ophthalmic Systems AG, Port, Switzerland; Sussex Eye Hospital, Brighton & New Cross Hospital, Wolverhampton, UK) under topical or local anesthesia. Trial surgeons were any ophthalmologists who routinely performed cataract surgery at their respective trial sites who had completed at least 10 supervised FLACS operations and had been certified by the manufacturers of Catalys or Ziemer. For FLACS, the laser was used to perform the capsulotomy and lens fragmentation. Laser arcuate keratotomy could be performed using the Catalys laser at the surgeon’s discretion. Detailed descriptions of the Catalys^19^^,^^20^ and Ziemer Z8^21^^,^^22^ use for cataract surgery have been published. All patients had planned implantation of a monofocal IOL (Alcon SN60WF, St. Ann’s Hospital, Moorfields; Rayner 970C Sussex Eye Hospital, Brighton; Johnson & Johnson Tecnis 1 ZCB00 New Cross Hospital, Wolverhampton). Standard phacoemulsification was performed as per local practice. Management of astigmatism was at the treating ophthalmologist’s discretion. Before randomization, the surgeon indicated whether he/she would use a toric lens if local NHS funding arrangements permitted or a limbal-relaxing incision for a manual patient or an astigmatic keratotomy for a laser case.

Postoperative care including eye drops was as per standard unit practice for cataract surgery. When the laser treatment could not be performed for whatever reason after randomization to FLACS (e.g., unable to dock, laser machine fault), patients received PCS.

### Outcomes

The primary outcome was unaided distance visual acuity (Early Treatment Diabetic Retinopathy Study logarithm of the minimum angle of resolution [logMAR] chart at a starting distance of 4 m)^23^ in the study eye at 3 months' follow-up.

Secondary outcomes were corrected distance visual acuity at 3 months in the study eye. Safety measures included intraoperative and postoperative complications,^24^ corneal endothelial cell count change, and refractive error (spherical equivalent) within 0.5 D and within 1.0 D of intended refractive outcomes. Health-related quality of life was measured by the EQ-5D-3 L questionnaire + vision bolt-on question (EQ-5DV)^25^ at 6 weeks and 3 months, and patient-reported vision health status was measured using the Catquest-9SF,^26^ a Rasch validated instrument at 6 weeks and 3 months.

Outcome measures are detailed in the trial protocol (version 4.0, 27 September 2016 available at https://fundingawards.nihr.ac.uk/award/13/04/46). For participants without complete postal questionnaire data, a telephone interview was done for additional clarification and completion of missing items. Staff performing outcome measures were all trained in their collection and masked to trial arm for trial postoperative assessments, including visual acuity, subjective refraction, corneal measurements, and endothelial cell count. After these measures had been completed, complications data were collected by patient medical notes review, for which masking was not possible. Additional secondary outcomes will be collected at 12 months postoperatively, including unaided distance visual acuity (UDVA), corrected distance visual acuity, patient-reported health, safety outcomes, and health economic analysis. This article reports the primary and secondary outcomes 3 months after surgery.

### Statistical Analysis

The trial was framed as a noninferiority design to demonstrate that visual acuity after laser-assisted cataract surgery is not inferior to that achieved after manual phacoemulsification cataract surgery. The noninferiority margin was based on a prespecified difference in mean UDVA of 0.1 logMAR (5 letters, or 1 line on the eye chart) that was considered to be clinically important to patients and ophthalmologists based on prior patient and public input to the trial design. Interpretation of the trial results is based on the 95% confidence interval (CI) for the difference between laser and manual surgery. If the 95% CI for the difference lies wholly to the left of the noninferiority margin, then we can conclude that laser surgery is not inferior to manual surgery. If the 95% CI for the difference lies wholly to the left of zero (i.e., the 95% CI excludes zero), then we can conclude that laser surgery is superior to manual surgery. We performed sequential testing of the noninferiority and superiority hypotheses.

We aimed to recruit at least 808 patients (404 per arm). The test–retest variability is reported as approximately 0.07 logMAR on letter-by-letter scoring.^27^^,^^28^ If there is truly no difference in mean logMAR between the 2 groups, then 432 patients (216 per group) would provide 90% power to be sure that a 95% 2-sided CI would exclude the prespecified noninferiority limit of 0.1 logMAR, assuming a common standard deviation of 0.32. The standard deviation is from the Royal College of Ophthalmologists’ National Ophthalmic Database UDVA data.^24^ However, although treatment is delivered on an individual basis, each patient cannot be assumed to generate independent information because they will be clustered within surgeons. To take account of clustering by surgeon (i.e., the variation between surgeons in the treatment effect), the sample size was increased by an inflation factor f = 1+(m−1)×p. Assuming a total of 16 surgeons contribute an average cluster size (m) of 50 patients and an estimated intraclass correlation (ICC) (p) of 0.012, this gives an f of 1.59. A total of 688 patients (344 per group) enabled the trial to take account of clustering by surgeon. To allow for an anticipated 15% dropout rate, the median age of patients undergoing cataract surgery in the United Kingdom is 77 years,^24^ and many have significant systemic comorbidities. Thus, the total sample size required was 808 patients. All primary and secondary analyses were conducted following the intention-to-treat principle retaining patients in the group to which they were randomly allocated irrespective of the treatment received.

A detailed Statistical Analysis Plan was approved before performing the statistical analysis (https://fundingawards.nihr.ac.uk/award/13/04/46). Missing data for the primary outcome were imputed using multiple imputation with chained equations with results combined using Rubin’s rules. Additional sensitivity analyses of the primary outcome were a per-protocol and a complete-case basis. Analysis of secondary outcomes was performed on complete cases only. All regression models included site and number of eyes eligible as covariates; surgeon was included in the models as random effects. The model for the primary outcome also adjusted for baseline habitual logMAR visual acuity values, and similar adjustments were made for any continuous secondary outcomes if a baseline value was recorded. Astigmatism at baseline (as measured by keratometry readings from Pentacam corneal topography) was included as a covariate in the analyses of visual acuity outcomes. Adjusted treatment effect estimates, 2-sided 95% CIs, and 2-sided *P* values are reported for each outcome measure.

### Trial Oversight

An independent trial steering committee provided oversight of the trial to safeguard the interests of participants, and an independent data monitoring committee had access to data by randomization arm.

### Role of the Funding Source

The trial was funded by the UK National Institute for Health Research Health Technology Assessment program. The National Institute of Health Research had input to the trial design through peer review of the funding proposal but had no role in data collection, data analysis, or writing of this report but had sight of the final version of the article before publication.

## Results

**Figure 1:**
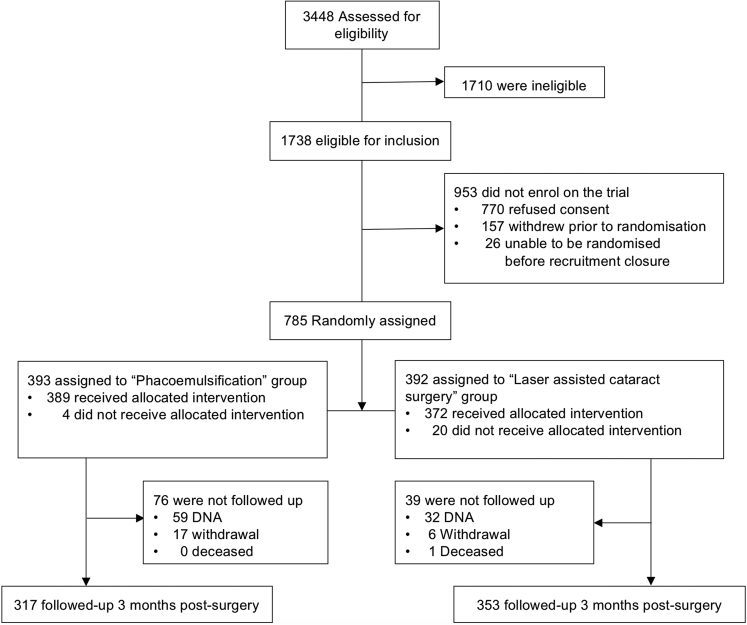
The FACT trial Consolidated Standards of Reporting Trials (CONSORT) flow diagram.

Of the 3448 patients assessed, we enrolled 785 participants between May 2015 and September 2017 (Moorfields: 653, Sussex Eye Hospital: 32, New Cross Hospital: 100) and randomly assigned 392 to FLACS and 393 to PCS (Fig 1).

The main reasons for exclusion (1710) were not sufficiently fluent in English for informed consent and trial questionnaire completion (564), postoperative refractive target outside ±0.5 D emmetropia (180), poor pupil dilation (176), and not willing to attend for follow-up (155). Of the 1738 patients eligible to participate, 770 declined to take part, 157 withdrew before randomization, and 26 were awaiting randomization at recruitment closure.

Forty major protocol deviations were identified: not receiving treatment according to randomization (25 participants [3.2%], 21 allocated to FLACS and 4 allocated to PCS) and not fulfilling refractive target eligibility criteria (15 participants, 10 allocated to FLACS and 5 allocated to PCS; Appendix shows further details, available at www.aaojournal.org). Overall, 353 (90%) of 392 participants allocated to FLACS and 317 (81%) of 393 participants allocated to PCS attended their follow-up visit at 3 months. Figure 1 shows the trial profile. Table 1 shows the trial population baseline characteristics by randomized group. Participant demographics and preoperative ocular biometric characteristics were similar. Analysis of toric IOL use by arm showed 22 toric lenses were used in the FLACS arm (369 monofocal, 1 data missing), and 19 toric lenses were used in the PCS arm (370 monofocal, 4 data missing). In total, 21 eyes (6%) that received FLACS had laser astigmatic keratotomies. Table 2 shows the postoperative results at 3 months by treatment arm. Table 3 shows intraoperative complications, and Table 4 shows postoperative complications by treatment arm. There were no significant differences between arms for any outcomes.

**Table 1:** Trial Population Baseline Characteristics by Randomized Group

Parameter	FLACS	PCS
Gender, male/female n, (%)	182 (46%)/210 (54%)	192 (49%)/201 (51%)
Previous cataract surgery (second eye cataract surgery in trial)	82 (21%)	72 (18%)
Right eye/left eye n, (%)	206 (53%)/186 (47%)	226 (57%)/167 (43%)
Age, y, (SD)	68 (10)	68 (10)
Ethnicity		
White n (%)	281 (72)	272 (69)
Mixed n (%)	3 (0.8)	7 (2)
Asian or Asian British n (%)	33 (8)	46 (12)
Black or Black British n (%)	57 (15)	52 (13)
Other ethnic groups n (%)	18 (5)	15 (4)
Not declared	0 (0)	1 (0.3)
Anterior chamber depth, mm (SD)	3.22 (0.41)	3.21 (0.39)
Axial length, mm (SD)	24.00 (1.49)	23.97 (1.47)
Preoperative corneal astigmatism, n (%)		
<0.75 D	194 (49)	177 (45)
0.75 to <2.0 D	163 (42)	184 (47)
≥2.0 D	34 (8.7)	29 (7.4)
Endothelial cell count	2640 (334)	2604 (348)
Macular thickness	249 (42)	249 (41)
Ocular copathology, n (%)	128 (33)	140 (36)
Catquest-9SF, score (SD)	0.62 (1.7)	0.52 (1.7)
EQ-5D-3L	0.79 (0.24)	0.78 (0.25)
EQ-5D-3L: visual analogue score	77.8 (18)	77.3 (18)
EQ-5DL-3L vision bolt on, n (%)		
I have no problems seeing	149 (38)	137 (35)
I have some problems seeing	127 (32)	114 (29)
I have extreme problems seeing	6 (1.5)	5 (1.3)
Missing, n (%)	110 (28)	137 (35)

D = diopters; FLACS = femtosecond laser-assisted cataract surgery; PCS = phacoemulsification cataract surgery; SD = standard deviation.

**Table 2:** Postoperative Results at 3 Months by Treatment Arm

Outcome	FLACS	PCS	Effect, FLACS vs. PCS (95% CI)	*P* Value
UDVA logMAR, imputed, mean (SD)	0.13 (0.23)	0.14 (0.27)	−0.01 (−0.05 to 0.03)	0.63
UDVA logMAR, complete case, mean (SD)	0.13 (0.23)	0.14 (0.26)	−0.01 (−0.04 to 0.03)	0.70
UDVA logMAR, per protocol, mean (SD)	0.13 (0.22)	0.14 (0.26)	−0.01 (−0.05 to 0.02)	0.54
CDVA logMAR mean (SD)	−0.01 (0.19)	0.01 (0.21)	−0.01 (−0.05 to 0.02)	0.34
SE refraction within ±0.5 D of target, n (%)	250/352 (71%)	224/316 (71%)	1.01 (0.72–1.41)	0.95
SE refraction within ±1.0 D of target, n (%)	327/352 (93%)	292/316 (92%)	1.08 (0.60–1.94)	0.80
Change in endothelial cell count, mean loss (SD)	242 (416)	200 (369)	47 (−3 to 97)	0.06
Catquest 9-SF, score, mean (SD)	2.30 (1.31)	2.27 (1.30)	0.07 (−0.13 to 0.28)	0.49
EQ-5D-3L index score mean (SD)	0.84 (0.23)	0.82 (0.25)	0.0002 (−0.03 to 0.03)	0.88
I have no problems seeing	235 (67%)	220 (68%)	-	-
I have some problems seeing	114 (32%)	100 (31%)	-	-
I have extreme problems seeing	3 (0.9%)	3 (0.9%)	-	-

CDVA = corrected distance visual acuity; CI = confidence interval; D = diopters; FLACS = femtosecond laser-assisted cataract surgery; logMAR = logarithm of the minimum angle of resolution; PCS = phacoemulsification cataract surgery; SD = standard deviation; UDVA = unaided distance visual acuity.

**Table 3:** Intraoperative Complications (n %)

	FLACS (n = 391)	PCS (n = 389)
≥1 intraoperative complications∗	11 (2.8%)	5 (1.3%)
Anterior capsule tear	3	2
Posterior capsule tear with vitreous loss	0	0
Posterior capsule tear, no vitreous loss	0	2
Zonular dialysis with or without vitreous loss	1	0
Intraoperative pupil constriction needing intervention	3	1
Dropped lens fragments	0	0
Suprachoroidal hemorrhage	0	0
Incomplete laser capsulotomy	4	NA

FLACS = femtosecond laser-assisted cataract surgery; NA = not available; PCS = phacoemulsification cataract surgery.

∗ Difference = 1.5%; 95% CI, −0.5 to 3.5, *P* = 0.13.

**Table 4:** Postoperative Complications by Treatment Arm

Postoperative Complications, n (%)	FLACS (n = 391)	PCS (n = 389)
≥1 postoperative complications∗	49 (12.5%)	44 (11.3%)
Postoperative anterior uveitis	34 (9.7%)	32 (8.2%)
Endophthalmitis	0 (0%)	0 (0%)
Macular edema	8 (2.0%)	7 (1.8%)
Retinal tear or detachment	1 (0.3%)	1 (0.3%)
Steroid response ocular hypertension	4 (1.0%)	3 (0.8%)
Medication allergy or intolerance	4 (1.0%)	3 (0.8%)
Corneal edema	4 (1.0%)	2 (0.5%)
Vitreous to wound	1 (0.3%)	1 (0.3%)

FLACS = femtosecond laser-assisted cataract surgery; PCS = phacoemulsification cataract surgery.

∗ Difference = 1.2%; 95% CI, −3.3 to 5.8, *P* = 0.60.

## Discussion

The results of the FACT trial are that PCS is as good as FLACS in terms of vision, patient-reported health, and safety outcomes at 3 months. We found no significant difference between groups for UDVA (the primary outcome) or any of the prespecified secondary outcomes.

Mean postoperative UDVA for FLACS was 0.13 logMAR versus 0.14 logMAR for PCS. These are similar to the 0.15 logMAR for both FLACS and PCS at 1 month postoperatively in the recent RCT from St. Thomas’ Hospital, United Kingdom, of 400 eyes of 400 patients.^16^ We found no difference between arms for health-related quality of life as measured by the EQ-5D-3L questionnaire and vision bolt-on question (EQ-5DV) or patient-reported vision status using Catquest–9SF, a Rasch-validated instrument. For refractive outcomes, 71% of both FLACS and PCS cases were within ±0.5 D target, and 93% of FLACS and 92% of PCS cases were within ±1.0 D target compared with 73% and 93% eyes being within ±0.5 D and ±1.0 D target in a recent large EUREQUO (European Registry of Quality Outcomes) analysis of 282 811 cataract surgeries.^29^ Comparative values from the St. Thomas’ Hospital RCT of FLACS versus PCS were 71% and 77% eyes, respectively, within ±0.5 D and 94% and 95% eyes within ±1.0 D.^16^

Overall, our complication rates were lower or comparable to previously published data from big datasets on cataract surgery outcomes.^24^ Specifically, the posterior capsule rupture rates were 0.0% for FLACS and 0.5% for PCS compared with a reported UK benchmark rate of 2.0%.^24^ The St. Thomas’ RCT found a statistically significant lower posterior capsule rupture rate in the laser-assisted arm, 0.0% versus 3.0%, respectively.^16^ Previously, there had been some concern over possible higher anterior capsule tear rates in laser-assisted cataract surgery due to the “postage-stamp” edge pattern after laser capsulotomy creation, with rates of 1.9% reported for laser capsulotomy compared with 0.1% for standard capsulorhexis in a comparative case series of 1626 surgeries.^30^ In our trial, anterior capsule tear rates were 0.8% (3/391) for laser capsulotomy compared with 0.5% (2/389) for standard capsulorhexis, and this did not reach statistical significance. In the St. Thomas’ RCT, the anterior capsule tear rate was 3.0% for FLACS and 1.5% for PCS, and again, this did not reach statistical significance. In view of the low event rates of both anterior and posterior capsule tears, meta-analysis of RCT outcomes is required to investigate this further.

### Study Limitations

This trial was designed to be sufficiently powered to detect important differences in vision and to minimize possible bias. It was publicly funded and designed to be representative in the context of a publicly funded national health service in the United Kingdom. Masking of the operating surgeon was not possible because of the surgery methodology, and although trial participants were not masked to their allocated arm, we do not believe this to be a significant source of bias in the outcome measures. Of note, we did observe a small difference in the 3-month follow-up rates for FLACS versus PCS, with 90% of FLACS cases attending compared with 80% for PCS. Participants who did not attend were contacted by identical methods to rebook within trial time scales, and an additional sensitivity analysis does not suggest a difference in the characteristics of those who were lost to follow-up. There is a possible surgical learning curve effect for FLACS, with all trial surgeons having performed hundreds to thousands of conventional phacoemulsification cataract surgeries compared with a minimum of 10 FLACS cases to meet trial surgeon eligibility. We have previously published data on the learning curve for FLACS and found that complications tended to occur within the first few cases;^31^ however, correspondence suggests the learning curve may include the first 100 cases for FLACS.^32^ Even if the FLACS learning curve is 100 cases, the complication rate in the FLACS arm is low, and so it is difficult to see how this would materially affect our findings. Another limitation is the majority of cases were recruited from a high-volume cataract day surgery unit (St. Ann’s, Moorfields Eye Hospital), and so this setup may not be fully representative of the setup in other areas of the United Kingdom. Trial recruitment (785 participants) was slightly below the planned 808 total; however, based on the prerecruitment power calculation, the 95% CI for the difference in visual acuity (−0.05 to 0.03) did not include our noninferiority margin of 0.1 logMAR,^27^ considered to be appropriate for cataract drug efficacy trials.^27^ The FACT trial was not powered to identify differences in complication rates, such as posterior capsule rupture, that happen infrequently. Additional meta-analysis of available evidence is required to investigate for possible differences in these infrequently occurring events.

In conclusion, the results of the FACT trial with 3-month postoperative data found that PCS is as good as FLACS in terms of vision, patient-reported health, and safety outcomes at 3 months. Longer-term outcomes in terms of clinical and cost-effectiveness are awaited. Additional RCT data and meta-analysis are required to further investigate possible differences between the surgical methods because of the low complication rates and apparent similar efficacy.
