# Supplementary material for: Femtosecond Laser-Assisted Cataract Surgery Versus Phacoemulsification Cataract Surgery (FACT): A Randomized Noninferiority Trial
Source: Ophthalmology. 2020 Aug;127(8):1012–9. doi: 10.1016/j.ophtha.2020.02.028 (PMC7397499; doi:10.1016/j.ophtha.2020.02.028)
Supplement: Appendix [file mmc1.docx]

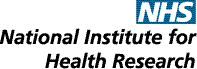

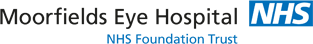


**
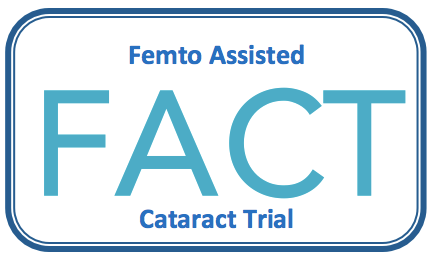
**

**A Randomised, Single Masked, Non-Inferiority Trial of Femtosecond Laser Assisted vs Manual Phacoemulsification Cataract Surgery for Adults with Visually Significant Cataract: the FACT trial**

| Version | 4.0 |
| --- | --- |
| Date | 27^th^ September 2016 |
| Sponsor | University College London (UCL) |
| Comprehensive Clinical Trials Unit Trial Adoption Group # | CTU/2012/046 |
| Trial registration | ISRCTN77602616 |
| REC | 14/LO/1937 |

**Authorisation: Chief Investigator**

| Name | Mark Wilkins |
| --- | --- |
| Role | Consultant Ophthalmologist, Moorfields Eye Hospital NHS Trust |
| Signature |  |
| Date |  |

**Authorisation: Sponsor/CCTU Director Representative & Senior Operations Staff**

| Name | Kate Maclagan |  |
| --- | --- | --- |
| Role | Clinical Project Manager, UCL CCTU |  |
| Signature |  |  |
| Date |  |  |

**Authorisation: Senior Statistics Staff**

| Name | Caroline Doré |
| --- | --- |
| Role | Head of Statistics, UCL CCTU |
| Signature |  |
| Date |  |

Table of Contents

[1 Administrative information 7](#_Toc413052056)

[1.1 Compliance 7](#_Toc413052057)

[1.2 Sponsor 7](#_Toc413052058)

[1.3 Structured trial summary 8](#_Toc413052059)

[*Inclusion criteria* 8](#_Toc413052060)

[*Exclusion criteria* 9](#_Toc413052061)

[1.4 Roles and responsibilities 10](#_Toc413052062)

[1.4.1 Protocol contributors 10](#_Toc413052063)

[1.4.2 Role of trial sponsor and funders 10](#_Toc413052064)

[1.4.3 Trial Team 11](#_Toc413052065)

[1.4.4 Trial Management Group 11](#_Toc413052066)

[1.4.5 Trial Steering Committee 11](#_Toc413052067)

[1.4.6 Data Monitoring Committee 12](#_Toc413052068)

[2 Summary Trial Flowchart 13](#_Toc413052069)

[3 Abbreviations 14](#_Toc413052070)

[4 Introduction 15](#_Toc413052071)

[4.1. Background and Rationale 15](#_Toc413052072)

[4.1.1 Explanation for choice of comparators](#_Toc413052073)

[4.2 Objectives 16](#_Toc413052074)

[4.3 Trial Design 17](#_Toc413052075)

[5 Methods 17](#_Toc413052076)

[5.1 Site Selection 17](#_Toc413052077)

[5.1.1 Study Setting 17](#_Toc413052078)

[5.1.2 Site/Principal Investigator Eligibility Criteria 17](#_Toc413052079)

[5.1.2.1 Principal Investigator’s (PI) Qualifications and Agreements 18](#_Toc413052080)

[5.1.2.2 Resourcing at site 18](#_Toc413052081)

[5.2 Site approval and activation 18](#_Toc413052082)

[5.3 Participants 18](#_Toc413052083)

[5.3.1 Eligibility Criteria 18](#_Toc413052084)

[5.3.1.1 Participant selection 18](#_Toc413052085)

[5.3.1.2 Participant Inclusion Criteria 19](#_Toc413052086)

[*Inclusion criteria* 19](#_Toc413052087)

[5.3.1.3 Participant Exclusion Criteria 19](#_Toc413052088)

[*Exclusion criteria* 19](#_Toc413052089)

[• Eyes with corneal ring and/or inlay implant(s), or severe corneal opacities, corneal abnormalities, significant corneal oedema or diminished aqueous clarity that is likely to obscure OCT imaging of the anterior lens capsule, 19](#_Toc413052090)

[• descemetocele with impending corneal rupture, 19](#_Toc413052091)

[• poor pupil dilation that is expected to require surgical iris manipultation, 19](#_Toc413052092)

[•subluxed crystalline lens. 19](#_Toc413052093)

[• Patient unable to give consent or unable to attend follow-up assessment. 19](#_Toc413052094)

[• Patient unable to be positioned for surgery. 19](#_Toc413052095)

[• Patient scheduled to undergo combined surgery e.g. cataract and trabeculectomy. 19](#_Toc413052096)

[• Any contraindications to cataract surgery. 19](#_Toc413052097)

[5.3.1.4 Eligibility Criteria for Surgeons 19](#_Toc413052098)

[5.3.1.5 Co-enrolment Guidance 20](#_Toc413052099)

[5.3.1.6 Screening Procedures and Pre-randomisation Investigations 20](#_Toc413052100)

[5.4 Interventions 21](#_Toc413052101)

[5.4.1 Arm A 21](#_Toc413052102)

[5.4.1.1 Manual Phacoemulsification cataract surgery 21](#_Toc413052103)

[5.4.2 Arm B 22](#_Toc413052104)

[5.4.2.1 Laser assisted Phacoemulsification cataract surgery 22](#_Toc413052105)

[5.4.3 Treatment Schedule 23](#_Toc413052106)

[5.4.4 Compliance and Adherence 23](#_Toc413052107)

[5.4.5 Concomitant Care 23](#_Toc413052108)

[5.5 Outcomes 24](#_Toc413052110)

[5.5.1 Primary Outcomes 24](#_Toc413052111)

[5.5.2 Secondary Outcomes 24](#_Toc413052112)

[5.6 Participant Timeline 25](#_Toc413052113)

[5.6.1 Early Stopping of Follow-up 26](#_Toc413052114)

[5.6.2 Participant Transfers 26](#_Toc413052115)

[5.6.3 Loss to Follow-up 26](#_Toc413052116)

[5.6.4 Trial Closure 26](#_Toc413052117)

[5.7 Sample Size 26](#_Toc413052118)

[5.8 Recruitment and Retention 27](#_Toc413052119)

[5.8.1 Recruitment 27](#_Toc413052120)

[5.8.2 Retention 27](#_Toc413052121)

[5.9 Assignment of Intervention 28](#_Toc413052122)

[5.9.1 Allocation 28](#_Toc413052123)

[5.9.1.1 Sequence generation 28](#_Toc413052124)

[5.9.1.2 Allocation concealment mechanism 28](#_Toc413052125)

[5.9.1.3 Allocation Implementation 28](#_Toc413052126)

[5.9.2 Masking 28](#_Toc413052127)

[5.9.3 Emergency Unmasking 28](#_Toc413052128)

[5.10 Data Collection, Management and Analysis 29](#_Toc413052129)

[5.10.1 Data Collection Methods 29](#_Toc413052130)

[5.10.2 Data Management 29](#_Toc413052131)

[5.10.4 Statistical Methods 30](#_Toc413052132)

[5.10.4.1 Statistical Analysis Plan 30](#_Toc413052133)

[5.10.4.2 Statistical Methods – Outcomes 30](#_Toc413052134)

[5.10.4.3 Additional Analyses - Subgroup 31](#_Toc413052135)

[5.10.5 Analysis Population and Missing Data 31](#_Toc413052136)

[5.10.6 Economic evaluation 31](#_Toc413052137)

[5.10.6.1 Within-trial analysis 31](#_Toc413052138)

[5.10.6.2 Model based analysis 32](#_Toc413052139)

[5.10.6.3 Health Economic Analysis Plan 32](#_Toc413052140)

[5.10.6.4 Additional analyses – Value of information 32](#_Toc413052141)

[5.11 Data Monitoring 32](#_Toc413052142)

[5.11.1 Independent Data Monitoring Committee 32](#_Toc413052143)

[5.11.2 Interim Analyses 32](#_Toc413052144)

[5.11.3 Data Monitoring for Harm 33](#_Toc413052145)

[5.11.3.1 Safety reporting 33](#_Toc413052146)

[5.11.3.3 Other Notifiable Adverse Events 34](#_Toc413052147)

[5.11.3.4 Investigator responsibilities relating to safety reporting 34](#_Toc413052148)

[5.11.3.5.1 Seriousness assessment 34](#_Toc413052149)

[5.11.3.5.2 Severity or grading of Adverse Events 34](#_Toc413052150)

[5.11.3.5.3 Causality 34](#_Toc413052151)

[5.11.3.5.4 Expectedness 35](#_Toc413052152)

[5.11.3.6 Notifications 36](#_Toc413052153)

[5.11.3.6.1 Notifications by the Investigator to CCTU 36](#_Toc413052154)

[5.11.3.6.2 CCTU responsibilities 37](#_Toc413052155)

[5.11.4 Quality Assurance and Control 37](#_Toc413052156)

[5.11.4.1 Risk Assessment 37](#_Toc413052157)

[5.11.4.2 Central Monitoring at CCTU 37](#_Toc413052158)

[5.11.4.3 On-site Monitoring 38](#_Toc413052159)

[5.11.4.3.1 Direct access to participant records 38](#_Toc413052160)

[5.11.4.4 Trial Oversight 38](#_Toc413052161)

[5.11.4.4.1 Trial Management Team 38](#_Toc413052162)

[5.11.4.4.2 Trial Management Group 38](#_Toc413052163)

[5.11.4.4.3 Independent Trial Steering Committee 38](#_Toc413052164)

[5.11.4.4.4 Independent Data Monitoring Committee 38](#_Toc413052165)

[5.11.4.4.5 Trial Sponsor 39](#_Toc413052166)

[6 Ethics and Dissemination 39](#_Toc413052167)

[6.1 Research Ethics Approval 39](#_Toc413052168)

[6.2 Competent Authority Approvals 39](#_Toc413052169)

[6.3 Other Approvals 39](#_Toc413052170)

[6.4 Protocol Amendments 39](#_Toc413052171)

[6.5 Consent or Assent 40](#_Toc413052172)

[6.6 Confidentiality 40](#_Toc413052173)

[6.7 Declaration of Interests 41](#_Toc413052174)

[6.8 Indemnity 41](#_Toc413052175)

[6.9 Finance 41](#_Toc413052176)

[6.10 Archiving 41](#_Toc413052177)

[6.11 Access to Data 41](#_Toc413052178)

[6.12 Ancillary and Post-trial Care 41](#_Toc413052179)

[6.13 Publication Policy 41](#_Toc413052180)

[6.13.1 Trial Results 41](#_Toc413052181)

[6.13.2 Authorship 42](#_Toc413052182)

[7 Ancillary Studies 42](#_Toc413052183)

[8 Protocol Amendments 42](#_Toc413052184)

[9 References 44](#_Toc413052185)

[10 Appendices 47](#_Toc413052186)

[10.1 Specular microscopy for obtaining central corneal endothelial cell density – Standard Operating Procedure 47](#_Toc413052187)

# 1 Administrative information

This document was constructed using the Comprehensive Clinical Trials Unit (CCTU) at UCL Protocol template Version 4. It describes the FACT trial, sponsored by UCL and co-ordinated by CCTU.

It provides information about procedures for entering participants into the trial, and provides sufficient detail to enable: an understanding of the background, rationale, objectives, trial population, intervention, methods, statistical analyses, ethical considerations, dissemination plans and administration of the trial; replication of key aspects of trial methods and conduct; and appraisal of the trial’s scientific and ethical rigour from the time of ethics approval through to dissemination of the results. The protocol should not be used as an aide-memoire or guide for the treatment of other patients. Every care has been taken in drafting this protocol, but corrections or amendments may be necessary. These will be circulated to registered investigators in the trial. Sites entering participants for the first time should confirm they have the correct version by contacting the FACT Trial Manager at CCTU.

CCTU supports the commitment that its trials adhere to the SPIRIT guidelines. As such, the protocol template is based on an adaptation of the Medical Research Council CTU protocol template (2012) and the Standard Protocol Items: Recommendations for Interventional Trials (SPIRIT) 2012 Statement for protocols of clinical trials (32). The SPIRIT Statement Explanation and Elaboration document (33) can be referred to, or a member of CCTU Protocol Review Committee can be contacted for further detail about specific items.

## 1.1 Compliance

The trial will be conducted in compliance with the approved protocol, the Declaration of Helsinki (2008), the principles of Good Clinical Practice (GCP) as laid down by the Commission Directive 2005/28/EC with implementation in national legislation in the UK by Statutory Instrument 2004/1031 and subsequent amendments, the UK Data Protection Act, and the National Health Service (NHS) Research Governance Framework for Health and Social Care (RGF). Agreements that include detailed roles and responsibilities will be in place between participating sites and CCTU.

Participating sites will inform CCTU as soon as they are aware of a possible serious breach of compliance, so that CCTU staff can fulfil requirements to report the breach if necessary to the CCTU Quality Management Group (QMG). A ‘serious breach’ is one that is likely to affect to a significant degree:

- The safety or physical or mental integrity of the subjects in the trial, or
- The scientific value of the trial.

## 1.2 Sponsor

UCL is the trial sponsor and has delegated responsibility for the overall management of the FACT trial to CCTU. Queries relating to UCL sponsorship of this trial should be addressed to the CCTU Director or via the trial team.

## 1.3 Structured trial summary

| Primary Registry and Trial Identifying Number | ISRCTN77602616 |
| --- | --- |
| Date of Registration in Primary Registry | 6^th^ January 2015 |
| Secondary Identifying Numbers |  |
| Source of Monetary or Material Support | National Institute of Health Research (HTA) |
| Sponsor | University College London with sponsor responsibilities delegated to CCTU. |
| Contact for Public Queries | FACT Trial Manager  UCL Comprehensive Clinical Trials Unit  Gower Street, London ,WC1E 6BT  0207 679 6163  ctu.FACT@ucl.ac.uk |
| Contact for Scientific Queries | Mr Mark Wilkins  Consultant Ophthalmologist  Moorfields Eye Hospital NHS Foundation Trust  162 City Rd, London  EC1V 2PD  [Mark.wilkins@moorfields.nhs.uk](mailto:Mark.wilkins@moorfields.nhs.uk)  020 7566 2473 |
| Public Title | FACT: Laser assisted versus standard ultrasound cataract surgery |
| Scientific Title | The FACT trial: A randomised, single masked, non-inferiority trial of **F**emtosecond laser **A**ssisted vs. manual phacoemulsification **C**a**T**aract surgery for adults with visually significant cataract |
| Countries of Recruitment | United Kingdom |
| Health Condition(s) or Problem(s) Studied | Patients with visually symptomatic cataract(s) |
| Intervention(s) | Arm A: Manual Phacoemulsification cataract surgery in the study eye  Arm B: Laser Assisted Phacoemulsification cataract surgery in the study eye  **NB. The study eye is defined as the first eye to undergo cataract surgery that is chosen by the patient in discussion with the surgeon. The allocated intervention will be used for patients having surgery on both eyes unless the patient expresses a wish not to receive the same intervention.** |
| Key Inclusion and Exclusion Criteria | *Inclusion criteria*   - Adults aged 18 or over with visually symptomatic cataract, one or both eyes. - Patients must be sufficiently fluent in English to provide informed consent and completion of the patient reported outcome measures. - Patients must be willing to attend for follow up at 3 and 12 months following surgery to the study eye. - Post-operative intended refractive target in the study eye is within ±0.5 dioptres emmetropia.  *Exclusion criteria*   - Eyes with corneal ring and/or inlay implant(s), or severe corneal opacities, corneal abnormalities, significant corneal oedema or diminished aqueous clarity that is likely to obscure OCT imaging of the anterior lens capsule - Descemetocele with impending corneal rupture - Poor pupil dilation that is expected to require surgical iris manipulation - Subluxed crystalline lens. - Patient unable to give consent or unable to attend follow-up assessment. - Patient unable to be positioned for surgery. - Patient scheduled to undergo combined surgery e.g. cataract and trabeculectomy. - Any contraindications to cataract surgery. - Any clinical condition which the investigator considers would make the patient unsuitable for the trial, including pregnancy.   These inclusion and exclusion criteria have been kept to a minimum to ensure the trial is representative of clinical practice. Pre-existing ocular comorbidity such as age related macular degeneration, diabetic retinopathy or glaucoma will not be reasons for exclusion.  If a participant is currently taking part in another clinical trial it will be at the discretion of the Chief Investigator to decide whether they may also be recruited to the FACT trial. Cases should be assessed on an individual basis. |
| Study Type | The trial is a pragmatic, randomised single masked non-inferiority trial to determine if the proposed advantages of laser assisted cataract surgery translates to real benefits for both patients and the NHS. |
| Date of First Enrolment | By 1^st^ May 2015 |
| Target Sample Size | 808 |
| Primary Outcome(s) | Unaided distance visual acuity (UDVA, logMAR) at 3 months following surgery on the study eye, measured using a standard ETDRS chart at a starting distance of 4 metres. |
| Key Secondary Outcomes | 1. Unaided distance visual acuity (UDVA) at 12 months following surgery in the study eye. 2. Corrected distance visual acuity (logMAR) at 3 and 12 months following surgery in the study eye (ETDRS logMAR chart at a starting distance 4 metres). 3. Ocular complications within 3 and 12 months of surgery in the study eye. A complication will be defined as any event that causes unintentional injury to an ocular structure, or requires additional treatment, or has a negative effect on a patient's health or eyesight. 4. Unaided and corrected visual acuity (using the ETDRS chart at a starting distance of 4 metres) and complications in the second eye (for those with bilateral cataracts), and with both eyes open at 3 and 12 months following surgery on the study eye 5. Proportion of patients with vision within 0.5 and within 1 dioptre of the intended refractive outcome at 3 and 12 months following surgery on the study eye. 6. Patient reported outcome: vision health status at 3, 6 and 12 months: Catquest-9SF (26) 7. Cost-utility analysis: Within-trial cost-effectiveness analyses at 12 months and expected cost- effectiveness over patient lifetime. 8. Corneal endothelial cell count (additional safety measure) in both eyes at 3 and 12 months following surgery in the study eye. |

## 1.4 Roles and responsibilities

These membership lists are correct at the time of writing; please see terms of reference documentation in the TMF for current lists.

### 1.4.1 Protocol contributors

| **Name** | **Affiliation** | **Role** |
| --- | --- | --- |
| Mark Wilkins | Moorfields Eye Hospital NHS Foundation Trust | Chief Investigator  Principal Investigator |
| Alex Day | UCL Institute of Ophthalmology | Sub- Principal Investigator |
| Susan Tebbs | UCL CCTU | Deputy Director of the UCL Comprehensive Clinical Trials Unit |
| Caroline Doré | UCL CCTU | Head of Statistics |
| Jennifer Burr | Uni. St Andrews | Co Applicant |
| Catey Bunce | Kings College London | Co Applicant |

### 1.4.2 Role of trial sponsor and funders

| **Name** | **Affiliation** | **Role** |
| --- | --- | --- |
| UCL |  | Trial Sponsor. |
| UCL CCTU | UCL | Specific functions have been delegated to the UCL CCTU by the Sponsor.  A Clinical Project Manager (CPM) at the UCL CCTU will oversee the Trial Manager and other operations staff who will be responsible for the day-to-day management of the trial and for providing support to the site staff. The CCTU staff will be involved in approaching sites, case report form development, database construction, protocol and participant information in collaboration with the Trial Management Group, and site initiation training. Training will be provided on all aspects of the trial, including the informed consent process and any safety reporting as well as aspects of good clinical practice which will be updated regularly. The CCTU staff will be responsible for routine and triggered monitoring visits, with oversight by the CPM, as well as auditing, if necessary, which will be provided by a member of CCTU staff independent of the trial. Feedback will be via a formal reporting process regarding trial progress and site quality. |
| NIHR HTA | NIHR | Trial Funder |

### 1.4.3 Trial Team

| **Name** | **Affiliation** | **Role and responsibilities** |
| --- | --- | --- |
| Mark Wilkins | Moorfields Eye Hospital NHS Foundation Trust | Chief Investigator  Principal Investigator |
| Alex Day | UCL Institute of Ophthalmology | Sub- Principal Investigator |
| Susan Tebbs | UCL CCTU | Deputy Director Comprehensive Clinical Trials Unit |
| Emilia Caverly | UCL CCTU | Clinical Project Manager |
| Felicia Ikeji | UCL CCTU | Trial Manager |
| Tola Erinle | UCL CCTU | Data Manager |

### 1.4.4 Trial Management Group

| **Name** | **Affiliation** | **Role and responsibilities** |
| --- | --- | --- |
| Mark Wilkins | Moorfields Eye Hospital NHS Foundation Trust | Chief Investigator |
| Alex Day | UCL Institute of Ophthalmology | Sub- Principal Investigator |
| Jennifer Burr | University of St Andrews | Co Applicant |
| Richard Wormald | Moorfields Eye Hospital NHS Foundation Trust | Co Applicant |
| Gary Rubin | UCL Institute of Ophthalmology | Co Applicant |
| Vincenzo Maurino | Moorfields Eye Hospital NHS Foundation Trust | Co Applicant |
| Catey Bunce | Kings College London | Co Applicant |
| Yvonne Sylvestre | UCL CCTU | Trial Statistician |
| Torsten Chandler | UCL CCTU | Junior Health Economist |
| Emilia Caverly | UCL CCTU | Clinical Project Manager |
| Felicia Ikeji | UCL CCTU | Trial Manager |
| Tola Erinle | UCL CCTU | Data Manager |

###

### 1.4.5 Trial Steering Committee

| Name | Affiliation | Role and responsibilities |
| --- | --- | --- |
| Professor David Spalton | London Clinic Eye Centre | Chair |
| Professor Ann Schilder | Royal National Throat, Nose and Ear Hospital | Non-independent member |
| Andrew Elders | Glasgow Caledonian University | Independent member |
| Larry Benjamin | Stoke Mandeville Hospital | Independent member |
| Horace Chung | N/A | Lay Member |

### 1.4.6 Data Monitoring Committee

| Name | Affiliation | Role and responsibilities |
| --- | --- | --- |
| Dr Chris Rogers | Bristol Royal Infirmary | Chair |
| Augusto Azuara-Blanco | Queen’s University Belfast | Independent Member |
| Emma Hollick | Kings College Hospital | Independent Member |

# 2 Summary Trial Flowchart

Non-study (current) patient cataract surgery pathway

Study patient pathway

(additional steps)

First seen in clinic and listed for cataract surgery

First seen in clinic and listed for cataract surgery.

More detailed measurement of your vision, eye and eye health: done same day or later depending on preference.

Standard ultrasound cataract surgery

Standard ultrasound cataract surgery

Routine patient follow-up at 2-4 weeks

Discharged

Listed for cataract surgery to other eye

Normal ultrasound (phacoemulsification) cataract surgery

Routine patient follow-up at 2-4 weeks

Discharged to local Optometrist

Routine patient follow-up at 2-4 weeks

Listed for cataract surgery to other eye

Cataract surgery on other eye: (same method as 1^st^ eye)

Routine patient follow-up at 2-4 weeks

**3 month study appointment**: repeat detailed measurement of vision, eye and eye health.

**12 month study appointment**: repeat detailed measurement of vision, eye and eye health.

6 week postal questionnaire

6 month postal questionnaire

Laser assisted cataract surgery

Discharged to local Optometrist

# 3 Abbreviations

| AE | Adverse Event | PCR | Posterior Capsule Rupture |
| --- | --- | --- | --- |
| AR | Adverse Reaction | PI | Principal Investigator |
| CI | Chief Investigator | QA | Quality Assurance |
| CRF | Case Report Form | QC | Quality Control |
| CCTU | Comprehensive Clinical Trials Unit | R&D | Research and Development |
| CDVA | Corrected Distance Visual Acuity | REC | Research Ethics Committee |
| CSRI | Client Service Receipt Inventory | SAE | Serious Adverse Event |
| EPT | Effective Phacoemulsification Time | SAP | Statistical Analysis Plan |
| ETDRS | Early Treatment Diabetic Retinopathy Study | SD | Standard Deviation |
| EU | European Union | SHO | Senior House Officer |
| FDA | (US) Food and Drug Administration | SPR | Specialist Registrar |
| FWA | Federal Wide Assurance | SSA | Site Specific Approval |
| GCP | Good Clinical Practice | TMF | Trial Master File |
| HTA | Health Technology Assessment | TMG | Trial Management Group |
| ICC | Intraclass correlation Coefficient | TMT | Trial Management Team |
| ICH | International Conference on Harmonisation | ToR | Terms of Reference |
| IDMC | Independent Data Monitoring Committee | UCL | University College London |
| IOL | Intraocular Lens | UDVA | Uncorrected Distance Visual Acuity |
| IRB | Institutional Review Board | VL | Vision Loss |
| ITT | Intention to Treat |  |  |
| LogMAR | Logarithm of the minimum angle of resolution |  |  |
| NHS | National Health Service |  |  |
| NIHR | National Institute for Health Research |  |  |

# 4 Introduction

## 4.1. Background and Rationale

Cataract is one of the leading causes of low vision in the UK. Cataract surgery is the most commonly performed operation by the NHS with over 310,000 procedures in 2011-12 at an approximate cost of £250 million. The current surgical method introduced over 20 years ago uses ultrasound (phacoemulsification) to help remove the cataract. Following surgery, 91% achieve a Snellen visual acuity of 6/12 or better (the minimum required for driving, 0.30 logMAR) and 46% achieve Snellen 6/6 or better (0.00 logMAR, 20/20 equivalent in the United States, perceived as normal vision.(2) The overall complication rate for cataract surgery is 7.4%.(4) Complications can affect recovery and some are serious and associated with poor long-term outcomes; eg. posterior capsule rupture (PCR)/ vitreous loss (VL) that occurs in ~1.9% cases. Of these one third of patients have complaints about their eye & vision 3.5 years after surgery (5). One in 5 cases need further surgery (6) and there is a 15 times higher risk of retinal detachment within 3 years.(7) The surgical learning curve is associated with complications, with a 3.7 and 1.6 times higher risk of PCR/VL for SHOs and SpRs respectively when compared to Consultants.(8) Other complications, the majority of which are less serious, may mean a longer operation and delayed healing with additional appointments and eye drops. Patients can be devastated when suffering a complication and due to the importance of vision for daily activities, can find even minor complications very distressing.

Laser systems are now available that can make the incisions, open the lens capsule and fragment the cataract in 1 minute, leaving only removal of lens fragments and insertion of the lens implant to be done by the surgeon.

There is increasing evidence to support advantages of laser assisted procedures with more accurate capsulotomy positioning, shape and size reported when compared to manual capsulorrhexis.(9–11) This is associated with better IOL centration (ensuring correct centering of the lens) (10–12) and less IOL tilt (12) with fewer internal higher order aberrations.(13) By using a laser to fragment the crystalline lens, less phacoemulsification (ultrasound) energy is subsequently required to complete its removal. Reductions of 70% to 96% of effective phacoemulsification time (EPT, ultrasound power) have been reported (14–16), with zero EPT being possible in 30% of operations in a recent series. (14) This study also reported a 36% lower endothelial cell loss in the laser assisted procedures compared to manual phacoemulsification. Data on complication rates in laser cataract surgery procedures has been reported in recent case series (17,18) with complication rates appearing favourable when compared to those from a large series of manual phacoemulsification (e.g. PCR rate of 0.31% in 1300 consecutive operations).(18)

The potential advantages are broad and include greater safety and better visual outcomes through greater precision and reproducibility. These systems are expensive but costs may be mitigated by greater efficiency (faster surgery), fewer complications, less repeat surgery and better outcomes. Both the NIHR and a recent review article (19) have identified the need for evidence comparing the safety and effectiveness of laser surgery to current practice.

The timing of this trial is critically important because laser assisted cataract surgery is being rapidly adopted worldwide despite the absence of any randomised controlled trials comparing its safety and efficacy to phacoemulsification, the current standard method. While the potential advantages of laser assisted surgery may be attractive to patients and surgeons, it is expensive and there are logistical and practical issues that need to be understood. The absence of good evidence for any advantage of laser assisted surgery has been highlighted in a recent review article in Eye (19) (the Scientific Journal of the Royal College of Ophthalmologists), by the NIHR Horizon Scanning Centre. (20) The topic is identified as a research priority by the national James Lind Alliance Sight Loss and Vision Priority Setting Partnership (see http://www.fightforsight.org.uk/sightlosspsp). Many theoretical advantages of laser cataract surgery have been reported in small clinical studies, and while estimates of overall safety have been reported from case series, there are no well-designed prospective randomised controlled trials comparing the effectiveness and safety of laser assisted to phacoemulsification cataract surgery. A Cochrane protocol (with search strategy) comparing laser assisted to manual phacoemulsification has been published.(21) The preliminary findings are 2 published randomised studies meeting the inclusion criteria, one powered to investigate endothelial cell loss (22) (damage to the inner layer of the cornea as a consequence of surgery); the other is a small RCT identified as being at risk of bias.(23) Neither report visual acuity outcomes between trial arms. A French 1,050 patient multi-centre randomised economic evaluation was identified from online trial registers at start date with completion planned for April 2016. (24)

Demand for laser cataract surgery already exists among NHS patients and some NHS trusts are already tendering for laser cataract surgery platforms. This trial will answer important questions about the potential introduction of laser cataract surgery platforms into NHS practice, and will also benchmark current surgical standards.

“It is already too early (for rigorous evaluation), until unfortunately, it’s suddenly too late.” Buxton’s law (25)

### 4.1.1 Explanation for choice of comparators

The standard treatment available for cataract surgery is manual phacoemulsification, therefore this was chosen as the comparator arm.

## 4.2 Objectives

The aim of this trial is to establish whether laser assisted cataract surgery is as good as or better than standard manual phacoemulsification in NHS cataract surgical units.

Computer controlled laser platforms can automate over half the steps currently completed by hand in cataract surgery. The theoretical advantages of laser assisted cataract surgery include greater safety, better vision and potentially lower long-term health costs through greater efficiency and safety. The trial is a large, pragmatic, randomised controlled trial, which will be able to determine if the postulated advantages of laser assisted cataract surgery translate to real benefits to both patients and the NHS.

Proposed advantages will be assessed by evaluating the following at 3 and 12 months post-surgery:

1) Visual acuity: uncorrected distance visual acuity (UDVA in the study eye, primary outcome at 3 months), and corrected distance visual acuity (CDVA, secondary outcome), using the ETDRS chart at a distance of 4 metres.

2) Patient reported outcome measures: vision health status using Catquest-9SF (26) questionnaires.

3) Ocular complications.

4) Cost-effectiveness.

An optometrist, masked to the surgical intervention received, will measure clinical outcomes. The type of surgery is not macroscopically discernible during the examination.

The trial will inform NHS service providers, commissioners, patients and Ophthalmologists about the effectiveness and cost-effectiveness of these two methods of cataract surgery and will help to guide the possible introduction of laser assisted cataract surgery in the NHS.

## 4.3 Trial Design

This is a pragmatic, randomised controlled non-inferiority trial to establish whether laser assisted cataract surgery is as good as or better than standard manual phacoemulsification in NHS cataract surgical units.

# 5 Methods

## 5.1 Site Selection

The trial sponsor has overall responsibility for site and investigator selection and has delegated this task to CCTU.

### 5.1.1 Study Setting

The trial will be conducted in secondary care in NHS cataract day surgery units in England. The primary site is the cataract day surgery unit at St. Ann’s Hospital, Tottenham, North London (Moorfields Eye Hospital NHS Foundation Trust) and there is the potential to add further sites.

The research sites selected will be NHS day care surgery units who see large numbers of patients for routine cataract surgeries and therefore have a sufficient pool of patients to be able to recruit successfully to a large randomised trial. In addition, sites will have access to the laser equipment and will reflect a realistic NHS scenario for this trial.

### 5.1.2 Site/Principal Investigator Eligibility Criteria

Once a site has been assessed as being suitable to participate in the trial, the trial team will provide them with a copy of this protocol.

To participate in the **FACT** trial, investigators and trial sites must fulfil a set of criteria that have been agreed by the **FACT** Trial Management Group (TMG) and that are defined below.

Eligibility criteria:

- A named clinician is willing and qualified to take Principal Investigator responsibility
- Suitably trained staff are available to recruit participants, enter data and collect samples
- Access to appropriate laser equipment to perform the surgery

Trial sites meeting eligibility criteria and accepted by the TMG as being suitable to recruit to the trial, will be issued with the **FACT** Trial Master File (TMF) documentation to use when applying for NHS Permissions or local institutional approval as applicable.

#### 5.1.2.1 Principal Investigator’s (PI) Qualifications and Agreements

The investigator(s) must be willing to sign a CCTU Clinical Trial Site Agreement to comply with the trial protocol (confirming their specific roles and responsibilities relating to the trial, and that their site is willing and able to comply with the requirements of the trial). This includes confirmation of appropriate qualifications, familiarity with both surgical procedures, agreement to comply with the principles of GCP, to permit monitoring and audit as necessary at the site, and to maintain documented evidence of all staff at the site that have been delegated significant trial related duties.

#### 5.1.2.2 Resourcing at site

The investigator(s) should be able to demonstrate a potential for recruiting the required number of suitable subjects within the agreed recruitment period (i.e. the investigator(s) regularly treat(s) the target population). They should also have an adequate number of qualified staff and facilities available for the foreseen duration of the trial to enable them to conduct the trial properly and safely.

Sites will be expected to complete a delegation of responsibilities log and provide staff contact details.

Sites should have sufficient data management resources to allow prompt data return to CCTU.

## 5.2 Site approval and activation

On receipt of the signed Clinical Trial Site Agreement, approved delegation of responsibilities log and staff contact details, written confirmation will be sent to the site PI. The Trial Manager or delegate will notify the PI in writing of the plans for site initiation. Sites will not be permitted to recruit any patients until a letter confirming site activation has been issued. The Trial Manager or delegate will be responsible for issuing this following the MHRA approved green light to recruit process.

The site must agree to conduct the trial in compliance with the protocol as agreed by the Sponsor and, by the regulatory authority(ies) (as appropriate), and which was given favourable opinion by the Research Ethics Committee (REC) and/or Institutional Review Board (IRB). The PI or delegate must document and explain any deviation from the approved protocol, and communicate this to the trial team at CCTU.

A list of activated sites may be obtained from the Trial Manager.

## 5.3 Participants

### 5.3.1 Eligibility Criteria

#### 5.3.1.1 Participant selection

There will be **NO EXCEPTIONS** (waivers) to eligibility requirements at the time of randomisation. Questions about eligibility criteria should be addressed PRIOR to attempting to randomise the potential participant.

The eligibility criteria for this trial have been carefully considered and are the standards used to ensure that only medically appropriate participants are entered. Participants not meeting the criteria should not be entered into the trial for their safety and to ensure that the trial results can be appropriately used to make future treatment decisions for other people with similar diseases or conditions. It is therefore vital that exceptions are not made to these eligibility criteria.

If a participant is currently taking part in another clinical trial, the decision to enrol them in the FACT trial will be at the discretion of the Chief Investigator. Any co-enrolment should be documented.

Participants will be considered eligible for enrolment in this trial if they fulfil all the inclusion criteria and none of the exclusion criteria as defined below.

#### 5.3.1.2 Participant Inclusion Criteria

### *Inclusion criteria*

- Adults aged 18 or over with visually symptomatic cataract in one or both eyes.
- Patients must be willing to attend for follow up at 3 and 12 months following surgery in the study eye.
- Patients must be sufficiently fluent in English for informed consent and completion of the health state questionnaires.
- Post-operative intended refractive target in the study eye is within ±0.5 dioptres emmetropia.

#### 5.3.1.3 Participant Exclusion Criteria

### *Exclusion criteria*

- Eyes with corneal ring and/or inlay implant(s), or severe corneal opacities, corneal abnormalities, significant corneal oedema or diminished aqueous clarity that is likely to obscure OCT imaging of the anterior lens capsule,
- Descemetocele with impending corneal rupture,
- Poor pupil dilation that is expected to require surgical iris manipulation,
- Subluxed crystalline lens.
- Patient unable to give consent or unable to attend follow-up assessment.
- Patient unable to be positioned for surgery.
- Patient scheduled to undergo combined surgery e.g. cataract and trabeculectomy.
- Any contraindications to cataract surgery.
- Any clinical condition which the investigator considers would make the patient unsuitable for the trial, including pregnancy.

#### 5.3.1.4 Eligibility Criteria for Surgeons

Surgeons will be required to have performed a minimum of 10 **complete** laser-assisted cataract surgery operations before they can act as a surgeon within the trial (for either arm). Ten cases is the number typically recommended by the manufacturers of the laser platforms for supervised training but certification may be given for less than 10. This will not overrule the requirement of 10 **completed** supervised laser assisted cataract operations for surgeons operating specifically for the trial, as stated above.

#### 5.3.1.5 Co-enrolment Guidance

The Principal investigator or Co-Investigator(s) at trial sites will be responsible for ascertaining whether the patient is currently taking part in a clinical trial. All patients will only be enrolled once into the trial, however one patient may have two surgeries if they require cataract surgery on both eyes. The investigator will be responsible for checking the patient notes against the screening/enrolment log at site prior to screening to ensure that the patient is not already enrolled in the trial.

Patients may not be enrolled in any other interventional trial without permission of the Chief Investigator. Co-enrolment in observational studies is acceptable.

#### 5.3.1.6 Screening Procedures and Pre-randomisation Investigations

Written informed consent to enter and be randomised into the trial will be obtained from participants, after explanation of the aims, methods, benefits and potential hazards of the trial and **BEFORE** any trial-specific procedures are performed. The only procedures that may be performed before obtaining written informed consent are those that would be performed on all patients in the same situation as usual standard of care.

- Patients will be referred to the cataract clinic by their Optometrist or GP for consideration for cataract surgery.
- All patients will be sent a standard hospital letter with a date and time for their clinic appointment.
  - At the same time, potential study participants will also be sent the FACT patient information sheet and a covering letter.
  - Patients will attend their clinic appointment for formal diagnosis of cataract and discussion of the risks vs benefits of cataract surgery by an Ophthalmologist or hospital Optometrist. Any patient who then consents for surgery will be given the option to discuss the FACT trial further, and will be given time to read/re-read the patient information sheet during their pre-operative assessment, all of which would be carried out as standard care prior to cataract surgery. The standard pre-operative assessment is typically performed the same day as the initial clinic appointment. If the patient wishes to take part in the trial, they will then be consented and the additional trial baseline tests performed (many cataract surgery patients are elderly and attendance on another day may not be practical for them). All patients will be given ample time to consider the trial and if they wish to consider it further, they will be asked to provide a contact number and their consent to be contacted again by telephone in 1-7 days’ time, where they will be offered a separate enrolment appointment if they want to take part in the trial.
- There may be some patients who attend the pre-operative assessment clinic that have not been sent the FACT PIS.
  - These patients will be given the FACT PIS when they arrive for their clinic appointment and will be given time to read the information. Once the patient is formally diagnosed by their Ophthalmologist or hospital Optometrist, and has decided to proceed with cataract surgery, patients will be given the option to discuss the FACT trial further, and will be given time to read the patient information sheet during their pre-operative assessment as described above. If the patient wishes to take part in the trial, they will be consented and the additional trial baseline tests performed as described above. Any patient who wishes to consider the trial further, will be asked for a contact number and their consent to be contacted again by telephone in 1-7 days time, when they will be offered a separate enrolment appointment if they want to take part in the trial.
  - No trial specific assessments will be carried out until the patient has had sufficient time to consider the information and has provided written informed consent to enter the trial. After this point, the patient will undergo any trial specific assessments.
  - We have deliberately allowed patients to decide to join the trial on the day of their clinic assessment as many are elderly and travel to attend a second appointment for trial consent and investigations may be inconvenient. However all patients will be given this option.
  - All “trial specific assessments” may be part of a patient’s routine pre-surgical workup regardless of trial participation as some Ophthalmologists may request these for further information prior to surgery. They are all non-contact measurements of vision or eye health or eye shape; and pose no risk above the inconvenience of undergoing them.
- All patients screened for the trial should be entered into the FACT screening log, and the reason for not entering the trial should be captured if this occurs.

## 5.4 Interventions

All patients will undergo cataract surgery, either manual phacoemulsification (arm A) or laser assisted phacoemulsification (arm B). For patients, who choose to have cataract surgery on both eyes, the same intervention (either arm A or B) will be offered.

Randomisation will be performed by a delegated member of the trial team on the day of surgery, and as close to the time of surgery as possible. Randomisation will be to either:

**Arm A: Manual phacoemulsification cataract surgery**

or

**Arm B: Laser assisted phacoemulsification cataract surgery**

### 5.4.1 Arm A

#### 5.4.1.1 Manual Phacoemulsification cataract surgery

Arm A is the current standard treatment.

All patients randomised to arm A will be treated with manual phacoemulsification cataract surgery.

This is the standard method of cataract surgery at participating trial sites. Typically this will involve topical or local anaesthesia (with or without concurrent sedation depending on patient preference). The eye to undergo cataract surgery will be dilated according to standard local unit practice.

All patients will be seen by a doctor or trained designated healthcare professional to ensure the appropriate surgical consents have been completed and the eye to be operated on marked (including corneal steep axis identification if required). The intraocular lens to be used will be selected from the biometry according to patient preferred refractive outcome.

Following local standard surgical checks (WHO guidelines), the patient will be transferred to the anaesthetic room or direct to the operating theatre where the intended anaesthetic is given. If a depot pellet has been used for dilation, this will be removed at this stage.

Phacoemulsification cataract surgery will then be performed according to local standard practice. This will typically involve a 2.20 – 2.75mm corneal incision, capsulorrhexis, hydrodissection, phacoemulsification, cortical clear up and intraocular lens implantation. Peri-operative antibiotics will be given again according to local standard practice. Surgeons may choose to treat corneal astigmatism by their preferred method including on axis surgery, additional corneal incisions (eg. limbal relaxing incisions or opposite clear corneal incisions); or by the use of a toric intraocular lens according to standard practice (typically >2.00 dioptres of regular corneal astigmatism). A plan for astigmatism will be made prior to randomisation. Post-operative care including post-operative eye drops will be as per standard unit practice for cataract surgery.

The surgery start time will be defined as application of antiseptic solution to the periocular region by the operating surgeon following the final patient check. The surgery end time as the removal of the surgical drape.

### 5.4.2 Arm B

#### 5.4.2.1 Laser assisted Phacoemulsification cataract surgery

Arm B is the interventional treatment.

All patients randomised to arm B will be treated with laser assisted phacoemulsification cataract surgery.

The patient will be prepared for surgery in the same way as for arm A.

The patient will be transferred to the laser room prior to the operating theatre, or to the operating theatre if the laser platform is sited there. If a depot pellet has been used for dilation, this will be removed now. Anaesthetic eye drops will be administered, the laser interface placed in contact with the eye with the patient lying down, the interface is docked to the laser platform, the eye is scanned by the integral guidance and the laser treatment delivered. The laser docking and treatment delivery will be in accordance with the laser manufacturer’s recommended procedure, as detailed in the relevant operating manual.

The laser will be used to perform capsulotomy (typically 4.8 to 5.5mm diameter), lens fragmentation and corneal incisions including astigmatic keratotomies. If a toric intraocular lens is to be used, astigmatic keratotomy will not be performed.

We expect the time required for positioning for laser docking, docking and laser delivery to take <10 minutes with the actual docking being an estimated 3 minutes, of which 1 minute is laser delivery.

To generate a more precise time for the laser procedure patients will be recorded by stopwatch using the defined start time as, surgeon attempting docking to the laser by applying the patient interface. The end time being defined as removal of the patient interface from the eye.

Following laser delivery, additional dilating drops will be administered (eg. cyclopentolate 1% and phenylephrine 2.5%).

The patient is then transferred to the operating theatre if applicable and the remainder of the care pathway is as per Arm A with the obvious exception that the surgical steps completed by laser do not need to be performed by the surgeon (this is expected to lead to a shorter operation time).

Where the laser treatment cannot be performed for whatever reason following randomisation to arm B (eg. unable to dock, laser machine fault etc), patients will undergo surgery in accordance to that for arm A.

Laser assisted cataract surgeries will only be performed by surgeons who have completed at least 10 supervised laser assisted cataract surgery operations.

A typical nomogram for the correction of astigmatism by intrastromal femtosecond laser astigmatic keratotomy will be provided to all trial surgeons, however they will be free to choose how they correct pre-existing corneal astigmatism, so as to reflect local standard practice.

### 5.4.3 Treatment Schedule

Patients will be added to a surgical list following enrolment and a letter will be sent to the patient with confirmation of the surgery date. Each patient will be randomised as close as possible to the time of surgery. Patients will attend for surgery typically between 4 and 8 weeks following enrolment. Surgery will occur only once, unless a patient has symptomatic cataract in both eyes, in which case the patient will return approximately 2-8 weeks later for surgery on the second eye. Surgery on the second eye will be performed according to the randomised allocation for the first eye, unless there is a specific patient preference not to do so.

### 5.4.4 Compliance and Adherence

It is not expected there will be any problems with compliance as the trial treatment is delivered on one occasion only.

Patients will be required to re-confirm their consent prior to randomisation on the day of surgery and a patient may change their mind about having surgery at this point. Investigators and staff at site should follow local procedures for ensuring informed consent for surgery has been given, and that all patients have had sufficient opportunity to ask questions about the surgery.

Patients will be required to comply with the follow up schedule, to complete postal questionnaires at 6 weeks and 6 months post-surgery, and attend clinic at 3 months and 12 months post-surgery following surgery to the study eye. Site staff will be responsible for booking these appointments and following up any patients who do not attend. No additional research visits are required for patients having surgery for both eyes.

### 5.4.5 Concomitant Care

For patients on pre-existing treatment for intraocular pressure control, or inflammatory eye disease, these will be continued as required for the treatment of the respective condition.

## 5.5 Outcomes

### 5.5.1 Primary Outcomes

*Primary outcome*

- Uncorrected distance visual acuity (UDVA, logMAR) at 3 months post-surgery in the study eye, measured using a standard ETDRS chart at a starting distance of 4 metres.

### 5.5.2 Secondary Outcomes

*Secondary outcomes (all time points relate to times after cataract surgery in the study eye)*

- Uncorrected distance visual acuity (UDVA) at 12 months following surgery on the study eye.
- Corrected distance visual acuity (logMAR) at 3 and 12 months following surgery on the study eye (ETDRS logMAR chart at a starting distance 4 metres).
- Ocular complications within 3 and 12 months of surgery on the study eye. A complication will be defined as any event that causes unintentional injury to an ocular structure, or requires additional treatment, or has a negative effect on a patient's health or eyesight.
- Uncorrected and corrected visual acuity (using the ETDRS chart at a starting distance of 4 metres) and complications in the second eye (for those with bilateral cataracts), and with both eyes open at 3 and 12 months following surgery on the study eye.
- Proportion of patients with vision within 0.5 and within 1 dioptre of intended refractive outcome (and second eye for those with bilateral cataracts) at 3 and 12 months following surgery on the study eye.
- Quality of life as measured by the EQ-5D-3L questionnaire+vision bolt-on question (EQ-5DV) at 6 weeks, 3, 6 and 12 months.
- Patient reported vision health status using Catquest-9SF (26) at 3, 6 and 12 months.
- Cost-utility analysis reported as the incremental cost-effectiveness ratio and cost-effectiveness acceptability curves.
- Corneal endothelial cell count (additional safety measure) in both eyes, at 3 and 12 months following surgery on the study eye.

## 5.6 Participant Timeline

**Figure 1: Schedule of Assessments**

|  | Standard pre-assessment (for study and non-study patients) | **Baseline** | **Randomisation** | **Follow up** | | | | |
| --- | --- | --- | --- | --- | --- | --- | --- | --- |
|  |  | Surgical Pre Assessment Visit | Randomisation and surgery | Standard non-study post-op appt | 6 weeks post-surgery (by post)  (ideally +/-14 days) | 1^st^ trial apt: 3 months post-surgery  (ideally +/-14  days) | 6 months post-surgery  (by post)  (ideally+/-14  days) | 2^nd^ trial apt: 12 months post-surgery  (ideally +/-14 days) |
| Visit Number | *1* | *1 or later* | 2 | *3* |  | *4* |  | *5* |
| Past medical and ocular history | X | X |  |  |  |  |  |  |
| Consent for cataract surgery | X |  |  |  |  |  |  |  |
| Informed Consent & eligibility screening |  | X |  |  |  |  |  |  |
| Identification of study eye |  | X |  |  |  |  |  |  |
| Visual acuity: UDVA, pinhole, +/- glasses (Snellen), each eye | X |  |  | X |  |  |  |  |
| Visual Acuity: UDVA^1^ & CDVA^2^ (logMAR) each eye and binocular |  |  |  |  |  | X |  | X |
| Visual acuity (LogMAR) with usual method of correction |  |  | X^7^ |  |  |  |  |  |
| Subjective refraction |  |  |  |  |  | X |  | X |
| Ocular biometry | X |  |  |  |  |  |  |  |
| Pentacam corneal topography | X^6^ | X |  |  |  | X |  | X |
| Optical coherence tomography (OCT)^3^ | X^6^ | X^8^ |  | X^6^ |  | X |  | X |
| Inclusion/Exclusion Criteria |  | X |  |  |  |  |  |  |
| Catquest-9SF questionnaire |  | X |  |  | X | X | X | X |
| EQ-5D-3L+vision bolt-on question (EQ-5DV) |  | X |  |  | X | X | X | X |
| CSRI^5^ |  | X |  |  |  | X | X | X |
| Endothelial cell count measurement | X^6^ | X^8^ |  |  |  | X |  | X |
| Surgery |  |  | X |  |  |  |  |  |
| Adverse Event Collection |  |  |  |  | X | X | X^4^ | X |

*^1^UDVA: uncorrected distance visual acuity. All visual acuity measures will use the standard ETDRS logMAR chart at 4 metres. At the baseline visit (usual pre-assessment clinic for cataract surgery) ETDRS will be the usual care visual acuity measure and thus can be taken prior to consent, ^2^CDVA: corrected distance visual acuity using subjective refraction result .^3^OCT will be measured as part of the standard pre-assessment for all patients. OCT will be repeated at 3 and 12 months. ^4^patient reported complications only. ^5^The Client Service Receipt Inventory (CSRI) is a questionnaire for collecting retrospective information about study patients’ use of health and social care services, accommodation and living situation, income, employment and benefits.(28) ^6^Some patients will have these tests performed at the standard pre-assessment visit, depending on the site local procedure for surgical pre-assessment. ^7^Current glasses or unaided. ^8^ baseline procedures which should be repeated if surgery is delayed for more than 3 months.*

### 5.6.1 Early Stopping of Follow-up

Participants will be followed up for 12 months following surgery on the study eye.

If a participant chooses to discontinue participation in the trial prior to surgery, they will be either listed for standard phacoemulsification cataract surgery and followed up in clinic, or discharged if they choose not to proceed with cataract surgery.

If a participant chooses to stop follow up, this view must be respected and the participant withdrawn from trial follow up. CCTU should be informed of the withdrawal in writing using the appropriate **FACT** documentation. Data already collected will be kept and included in analyses according to the intention-to-treat principle for all participants who stop follow up early. If a patient is only willing to return for one follow up visit, then this should be the 3 month visit.

### 5.6.2 Participant Transfers

If a participant moves from the area making continued follow up at their consenting centre difficult, every effort should be made to arrange for them to be followed at another participating trial centre. Written consent should be taken at the new centre and a copy of the participant’s CRFs provided to the new centre. Responsibility for the participant remains with the original consenting centre until the new consent process is complete.

### 5.6.3 Loss to Follow-up

Patients who are temporarily lost to follow up (i.e. they miss or delay one or more follow-up visits) will be able to resume their participation in the trial by keeping their remaining appointments as close as possible to the schedule defined in the protocol. All participants will be asked to provide contact details for, and consent to contact where necessary, a “best alternative contact” such as a relative or close friend.

Every effort will be made to maintain contact with all patients, however, if a patient misses the 3 and 12 month follow-up appointments and is not contactable by any means, they will be deemed to be lost to follow up.

### 5.6.4 Trial Closure

The end of the trial will be defined as when the last participating patient has attended their 12 month follow up visit, all data is complete and all data queries have been resolved.

The REC will be notified within 90 days of the end of the trial. A summary report of the research will be sent to the REC within 12 months of the end of the trial.

## 5.7 Sample Size

The primary clinical outcome is uncorrected visual acuity in the study eye on the ETDRS logMAR chart at a starting distance of 4 metres at 3 months following surgery, ascertained by an optometrist masked to the trial group. A change in visual acuity of 1 line of the chart is considered to be clinically important, one logMAR line is 5 letters (each letter is 0.02 logMAR) and the test-retest variability is reported as 0.07 logMAR. If there is truly no difference in mean logMAR between the two groups, then 432 patients (216 per group) would provide 90% power to be sure that a 95% two sided confidence interval would exclude the non-inferiority limit of 0.1 logMAR, assuming a common standard deviation (SD) of 0.32. The SD is from the Royal College of Ophthalmologists’ National Ophthalmic Database of unaided vision 3 months following cataract surgery (n= 20,155).

However, although treatment is delivered on an individual basis, each patient cannot be assumed to generate independent information since they will be clustered within surgeons. To take account of clustering by surgeon (i.e. the variation between surgeons in the treatment effect) the sample size must be increased by an inflation factor f = 1 + (m-1)*p. Assuming a total of 16 surgeons contribute and an average cluster size (m) of 50 (patients/surgeon) and an estimated ICC (p) of 0.012, this yields an f of 1.59. A total of 688 patients (344 per group) would enable the trial to take account of clustering by surgeon. To allow for an anticipated 15% dropout rate (the mean age of patients undergoing cataract surgery is 75 years old and many have significant systemic comorbidities) the total sample size required is 808 patients (404 per group). The sample size calculation was conducted using Stata/IC version 12.1 (StataCorp, College Station, TX, USA. The IDMC will be asked to review all the assumptions used for the sample size calculation before the end of recruitment.

## 5.8 Recruitment and Retention

### 5.8.1 Recruitment

We aim to recruit 808 patients in total (404 in each arm) over the 18 month recruitment period.

Patients will be recruited via NHS sites as described in section 5.1.1. Screening, recruitment and randomisation will be undertaken by qualified individuals at site, and this will be documented on the site delegation log. Individuals taking consent will have received appropriate training.

The trial will start with an internal pilot of recruitment at St Ann’s Hospital, London for the first 6 months. We expect to recruit 30 patients during the 1^st^ month and 34 patients per month thereafter. During the internal pilot phase we will identify and investigate any barriers to recruitment before opening recruitment out to additional site(s).

Screening logs, recruitment rates and dropout rates will be reviewed at monthly meetings of the Trial Management Group. Any barriers to recruitment will be investigated and mechanisms put in place to correct them. Failure to meet our target recruitment of 200 patients during the internal pilot will trigger submission of an action plan to HTA, including adding further trial site(s) if necessary, to ensure the target is met.

It is expected that St Ann’s Hospital will recruit approximately 75% of the total required, leaving the additional 25% to be recruited by additional trial site(s).

### 5.8.2 Retention

The follow up period for the FACT trial is for 12 months. Participants will be required to attend two clinic visits at 3 and 12 months and will also need to complete trial questionnaires that will be posted at 6 weeks and 6 months.

Completion and return of the questionnaires at 6 weeks and 6 months will provide a prompt for the trial team to remind the patient of their next visit. If the participant does not return the questionnaire at these visits, a member of the trial team will phone the patient to remind them to post the questionnaires or to bring them at their next visit. This contact will also be used as an additional reminder of the date of the next follow up visit.

## 5.9 Assignment of Intervention

### 5.9.1 Allocation

#### 5.9.1.1 Sequence generation

Patients will be randomised in a 1:1 ratio to either surgical procedure via the Sealed Envelope.com website. Sealed Envelope is a randomisation service provider that provides a proven, reliable and centralised randomisation system. The system will be custom designed to the trial requirements.

This will use minimisation with a random element with stratification by a) treatment centre, b) surgeon, and c) whether in the local clinician’s opinion, the patient will require surgery on one or both eyes.

A random trial arm allocation will be computer generated. Sealed Envelope will provide the randomised treatment for each participant.

#### 5.9.1.2 Allocation concealment mechanism

On the day of surgery and as close to the time of surgery as possible, delegated staff at site will enter the patient’s initials, gender, date of birth, date of consent, eligibility criteria fulfilment, centre, surgeon and whether the patient requires surgery in one or both eyes into the SealedEnvelope.com secure website, which will then allocate the randomised treatment. The treatment allocation will not be concealed to the investigator given this is not a masked trial. Usernames and passwords for Sealed Envelope will be provided to site staff during the site activation procedure.

#### 5.9.1.3 Allocation Implementation

The responsibility for enrolling and randomising participants into the trial lies with the Principal Investigator and staff at site.

Individuals at participating centres will be provided with a secure login to the sealedenvelope.com website, according to a delegation of responsibilities log. The users will be required to log into the website and answer eligibility questions before entering stratification data and being permitted to randomise. The randomisation result will be shown directly online, with an email confirmation to the user and also to the Trial Manager.

### 5.9.2 Masking

Due to the nature of the intervention, neither the trial participants nor the treating clinician will be masked to the treatment allocation.

To ensure masking of the outcomes data, visual acuity and refraction at 3 and 12 month follow up visits will be performed by a trial Optometrist who will be masked to the treatment allocation. After these measures have been completed, complications data will be collected by patient medical notes and reviewed by an unmasked delegated member of the trial team and entered on the Case Record Forms (CRFs). Each site will be responsible for putting procedures in place to ensure the Optometrist is kept masked at all times, and will remind each patient not to reveal their treatment to the trial Optometrist at these two visits.

### 5.9.3 Emergency Unmasking

As the trial participants and unmasked delegated site staff will have access to the treatment allocation, emergency unmasking will not be necessary.

## 5.10 Data Collection, Management and Analysis

### 5.10.1 Data Collection Methods

Each participant will be given a unique trial Participant Identification Number (PIN). Data will be collected at the time-points indicated in the Trial Schedule (Table 1).

Data will be collected by the trial sites using paper Case Record Forms (CRFs) and securely transferred to CCTU. The data will be entered into the database by a member of the **FACT** trial team and stored on secure servers based at UCL. Training on paper CRF completion and storage for site staff listed on the delegation of responsibilities log will be provided at the site initiation meeting(s).

Data collection, data entry and queries raised by a member of the **FACT** trial team will be conducted in line with the CCTU Standard Operating Procedure (SOP) and trial specific Data Management Plan.

Identification logs, screening logs and enrolment logs will be kept at the trial site in a locked cabinet within a secured room.

Clinical trial team members will receive trial protocol training. All data will be handled in accordance with the Data Protection Act 1998.

### 5.10.2 Data Management

Data will be entered in the approved **FACT** database by a member of the **FACT** trial team at CCTU and protected using established CCTU procedures.

Participants will be given a unique trial Participant Identification Number (PIN). Data will be entered under this identification number onto the central database stored on the servers based at CCTU. The database will be password protected and only accessible to members of the **FACT** trial team at CCTU, and auditors if requested. The servers are protected by firewalls and are patched and maintained according to best practice. The physical location of the servers is protected by CCTV and security door access.

The database and coding frames have been developed by the Clinical Trial Manager in conjunction with CCTU. The database software provides a number of features to help maintain data quality, including; maintaining an audit trail, allowing custom validations on all data, allowing users to raise data query requests, and search facilities to identify validation failure/ missing data.

After completion of the trial the database will be retained on the servers of UCL for on-going analysis of secondary outcomes.

The identification, screening and enrolment logs, linking participant identifiable data to the pseudononymised Participant Identification Number, will be held locally by the trial site. This will either be held in written form in a locked filing cabinet or electronically in password protected form on hospital computers. After completion of the trial the identification, screening and enrolment logs will be stored securely by the sites for 5 years unless otherwise advised by CCTU.

### 5.10.4 Statistical Methods

#### 5.10.4.1 Statistical Analysis Plan

The primary analysis will be conducted following the intention to treat principle where all randomised patients are analysed in their allocated group whether or not they receive their randomised treatment. Baseline characteristics will be summarised for each treatment groups. Continuous data will be summarised using means and standard deviations, if data appear Gaussian, or medians and interquartile ranges. Binary data will be reported as proportions and percentages.

All statistical tests will use a 2-sided P value of 0.05 unless otherwise specified. All confidence intervals presented will be 95% and two sided. A detailed statistical analysis plan will be developed for approval by the Trial Steering Committee and review by the Independent Data Monitoring Committee and finalised before the first substantive statistical analysis. All statistical analyses will be performed using Stata (StataCorp, College Station TX, USA).

#### 5.10.4.2 Statistical Methods – Outcomes

The primary outcome is uncorrected distance visual acuity at 3 months following surgery on the study eye, measured using an ETDRS chart at a starting distance of 4 metres. Mean UDVA will be reported together with a two-sided 95% confidence interval. This will be compared between treatment groups using regression analysis adjusting for baseline habitual logMar^[[1]](#footnote-2)^ visual acuity and the randomisation stratifiers (treatment centre, surgeon, and whether or not patients have one or both eyes eligible). We will include trial site in our regression models as a fixed effect and surgeon as a random effect. If the upper end of the 95% CI for the difference between means does not cross the non-inferiority limit of 0.1 logMAR, then laser surgery will be regarded as non-inferior. If the mean difference is negative and its 95% CI lies wholly to the left of zero, then we can conclude that laser surgery is superior to manual surgery. We will perform sequential testing of the non-inferiority and superiority hypotheses.

Secondary continuous outcomes such as uncorrected distance visual acuity at 12 months, corrected distance visual acuity and patient reported outcome measures Catquest-9SF will be analysed in a similar fashion. The percentage of study eyes experiencing adverse events (e.g. posterior capsule tears, dropped lens) in the two groups will be compared using Fisher’s exact tests.

In addition regression analyses will be performed for continuous outcomes such as corrected distance visual acuity at 12 months for the study eye, adjusting for baseline habitual logMar visual acuity and the randomisation stratifiers (treatment centre, surgeon, and whether or not patients have one or both eyes eligible). Baseline habitual logMar visual acuity is only collected for the study eye and therefore distance visual acuity outcomes for the fellow eye, whether they are unaided or corrected, will only be adjusted for the randomisation stratifiers.

Pearson’s correlation coefficients (or Spearman’s rank correlation coefficients, depending on the distribution of the data) will be used to assess the relationships between the continuous objective and subjective outcome measures.

#### 5.10.4.3 Additional Analyses - Subgroup

Planned subgroup analyses will be conducted to investigate possible interactions between treatment effects and whether or not surgery was required on both eyes, with separate estimates and confidence intervals being reported for such patients. We will also investigate a possible interaction between treatment effect and trial site as a pre-specified subgroup analysis.

### 5.10.5 Analysis Population and Missing Data

While there is no planned research visit between surgery and the postoperative visit at 3 months, it is likely that patients will attend standard NHS visits following surgery. Visual acuity data will therefore be extracted from patient records for all patients and used in the multiple imputation model for the primary outcome for patients who do not attend for 3 months follow-up.

Missing data will be dealt with using multiple imputation by chained equations. Results will be combined using Rubin’s rules. Data will be assumed to be missing at random (MAR), in essence the data available for patients before they drop out will be used to predict the endpoint. The imputation will be performed following a pre-specified procedure and conducted separately by trial group. Reasons for missingness may be important and these will be investigated using logistic regression of covariates on an indicator of missingness. Sensitivity analysis will investigate the validity of the missing at random assumption.

### 5.10.6 Economic evaluation

We will undertake a detailed analysis of the costs and the cost-utility of laser assisted phacoemulsification cataract surgery compared with manual phacoemulsification cataract surgery (standard care). The analyses will conform to accepted economic evaluation methods (eg NICE methods guidance). All costs will be assessed from the perspective of the NHS and personal social services. We will estimate cost and cost-utility a) for the ‘within-trial’ period, based on the clinical and health related quality of life results at baseline and follow-up, and b) over the expected lifetime of the patients. We have selected the EQ-5DV as the most appropriate instrument for use in this population.

#### 5.10.6.1 Within-trial analysis

The units of outcome for the within-trial cost-effectiveness analyses will be the incremental cost per unit change in the uncorrected distance visual acuity (UCVA) in the study eye and the incremental cost per quality-adjusted life year (QALY) gained. QALYs will be calculated based on the responses to the EQ-5D-3L+vision bolt-on question (EQ-5DV) collected at baseline and follow-up. Patient-specific utility profiles will be constructed assuming a linear change in utility values measured using the EQ-5DV questionnaire at baseline and six week, three month, six month and 12 month follow-up time points. Utility estimates will be calculated according to the area under the curve approach, adjusting for baseline differences in patients in the trial arms if necessary. Missing EQ-5DV and resource use data will be addressed using appropriate statistical methods in consultation with the trial statistician.

#### 5.10.6.2 Model based analysis

In the lifetime model, cost-effectiveness will be calculated in terms of the incremental cost per QALY gained. A review of previous cost-effectiveness and cost-utility analyses will be conducted to identify any existing modelling work that may be drawn on for developing the model structure and informing model parameters. The specific details of the data required to populate the model will be determined following the development of the model structure. We will undertake deterministic (one-, two- and multi-way) and probabilistic sensitivity analysis, the latter assuming appropriate distributions and parameter values that will also be used to construct cost-effectiveness acceptability curves.

#### 5.10.6.3 Health Economic Analysis Plan

All analyses will be undertaken within a Bayesian framework. Methods for conducting economic evaluation using clinical trial data will be applied following O’Hagan and Stevens (30) and O’Hagan, Stevens and Montmartin. (31) Monte Carlo simulation methods will be used to construct a cost-effectiveness acceptability curve, based on the expected net benefit statistic, to estimate the probability that the intervention is cost-effective for a range of values of societal willingness to pay per QALY. We will also subject the results to extensive deterministic (one, two, and multi-way) sensitivity analysis.

Cost components included in the analysis will consist of (but not necessarily be limited to) the cost of surgery for both arms of the trial, complications arising from surgery, all relevant diagnostic investigations, revision surgery where necessary, hospital length of stay, outpatient attendances, hospital readmissions, primary care contacts, A&E attendances, and prescribed medications. The volume of resource use for each cost component will be measured directly from trial CRFs. Unit costs will be taken from standard published sources where possible. Where published unit costs are not available site specific unit costs will be obtained as required.

#### 5.10.6.4 Additional analyses – Value of information

No value of information analysis is planned at present.

## 5.11 Data Monitoring

### 5.11.1 Independent Data Monitoring Committee

Further details of the roles and responsibilities of the Independent Data Monitoring Committee (IDMC), including membership, relationships with other committees, decision making processes, and the timing and frequency of interim analyses (and description of stopping rules and/or guidelines where applicable) are described in detail in the **FACT** IDMC Terms of Reference (ToR).

### 5.11.2 Interim Analyses

No formal interim analysis is planned, but reports concerning patient safety and key efficacy outcomes will be prepared for review by the Independent Data Monitoring Committee who may request an interim analysis if a report raises concern.

The IDMC will also be asked to review all the assumptions used for the sample size calculation before the end of recruitment.

### 5.11.3 Data Monitoring for Harm

#### 5.11.3.1 Safety reporting

Definitions of harm of the EU Directive 2001/20/EC Article 2 based on the principles of ICH GCP apply to this trial.

Table 2: Adverse Event Definitions

| **Adverse Event (AE)** | Any untoward medical occurrence in a study participant which does not necessarily have a causal relationship with the treatment under study e.g. abnormal laboratory findings, unfavourable symptoms or diseases. |
| --- | --- |
| **Serious Adverse Event (SAE)** | Any AE that :   - results in death - is life threatening* - requires hospitalisation or prolongs existing hospitalisation** - results in persistent or significant disability or incapacity - is a congenital anomaly or birth defect - or is another important medical condition*** |
| **Suspected Unexpected Serious Adverse Event** | A serious adverse event, the nature or severity of which **is not consistent** with the known potentially **expected** events associated with the applicable trial treatment. The event is evaluated as having a possible, probable or definite relationship to a trial treatment **and** is unexpected for that trial treatment. |
| * the term life threatening here refers to an event in which the patient is at risk of death at the time of the event; it does not refer to an event that might hypothetically cause death if it was more severe (eg a silent myocardial infarction)  ** Hospitalisation is defined as an in-patient admission, regardless of length of stay, even if the hospitalisation is a precautionary measure for continued observation. Hospitalisation for pre-existing conditions (including elective procedures that have not worsened) do not constitute an SAE  *** Medical judgement should be exercised in deciding whether an AE is serious in other situations. | |

Adverse events include:

- an exacerbation of a pre-existing illness
- an increase in the frequency or intensity of a pre-existing episodic event or condition
- a condition (regardless of whether PRESENT prior to the start of the trial) that is DETECTED after surgery. (This does not include pre-existing conditions recorded as such at baseline.)
- continuous persistent disease or a symptom present at baseline that worsens following surgery

Adverse events do NOT include:

- Medical or surgical procedures: the condition that leads to the procedure is the adverse event
- Pre-existing disease or a condition present before treatment that does not worsen
- Hospitalisation where no untoward or unintended response has occurred eg elective cosmetic surgery

#### 5.11.3.3 Other Notifiable Adverse Events

All cases of posterior capsule rupture with vitreous loss including dropped nucleus will be notified to the CCTU and Chief Investigator within 48 hours of the time of surgery.

Any laser errors or faults resulting in non-delivery of the laser to perform the intended steps will be notified to the CCTU and Chief Investigator within 48 hours of the time of surgery.

Any pregnancies during the trial will be notified to the CCTU immediately the site are aware. Patients should continue to attend follow up visits until birth. Patients should be encouraged to attend follow up visits until birth. A pregnancy outcome form must be submitted to CCTU and will be queried if not received within 10 months of the pregnancy being reported.  Failure to send a pregnancy outcome form may result in a triggered on site monitoring visit.

#### 5.11.3.4 Investigator responsibilities relating to safety reporting

All non-serious AEs, whether expected or not, should be recorded in the patient’s medical notes and reported in the toxicity (symptoms) section of the Follow-up Form and sent to CCTU within 4 weeks of the study visit. TSAEs should be notified to CCTU immediately the investigator becomes aware of the event (in no circumstance should this notification take longer than 24 hours).

##### 5.11.3.5.1 Seriousness assessment

When an AE occurs, the investigator responsible for the care of the participant must first assess whether or not the event is serious using the definition given in Table 1. If the event is classified as ‘serious’ then an SAE form must be completed and CCTU notified immediately.

##### 5.11.3.5.2 Severity or grading of Adverse Events

The severity of all AEs (serious and non-serious) in this trial should be graded using the most current version of the Common Terminology Criteria for Adverse Events (CTCAE) of the National Cancer Institute (NCI) (version 4.0, 3, June 2010 at the time of writing this Protocol).

Severity is based on a score between 1 and 5 as follows:

- **Grade 1** - Mild; asymptomatic or mild symptoms; clinical or diagnostic observations only; intervention not indicated.
- **Grade 2** - Moderate; minimal, local or non-invasive intervention indicated; limiting age-appropriate instrumental Activities of Daily Living (ADL).
- **Grade 3** - Severe or medically significant but not immediately life-threatening; hospitalisation or prolongation of hospitalisation indicated; disabling; limiting self-care ADL.
- **Grade 4** - Life-threatening consequences; urgent intervention indicated.
- **Grade 5** - Death related to AE.

##### 5.11.3.5.3 Causality

The investigator must assess the causality of all serious events in relation to the trial therapy using the definitions in Table 2.

**Table 2: Causality definitions**

| Relationship | Description | Event type |
| --- | --- | --- |
| Unrelated | There is no evidence of any causal relationship | Unrelated SAE |
| Unlikely to be related | There is little evidence to suggest that there is a causal relationship (eg the event did not occur within a reasonable time after administration of the trial intervention). There is another reasonable explanation for the event (eg the participant’s clinical condition or other concomitant treatment) | Unrelated SAE |
| Possibly related | There is some evidence to suggest a causal relationship (eg because the event occurs within a reasonable time after surgery). However, the influence of other factors may have contributed to the event (eg the participant’s clinical condition or other concomitant treatment) | Related SAE |
| Probably related | There is evidence to suggest a causal relationship and the influence of other factors is unlikely | Related SAE |
| Definitely related | There is clear evidence to suggest a causal relationship and other possible contributing factors can be ruled out. | Related SAE |

##### 5.11.3.5.4 Expectedness

If there is at least a possible involvement of the trial intervention (including any comparators), the investigator and sponsor must assess the expectedness of the event. An unexpected adverse event is one that is not reported in the current literature, or one that is more frequently reported or more severe than previously reported. See below for a list of expected complications associated with the procedures being used in this trial.

Expected intraoperative complications for **both** arms include all those common to phacoemulsification cataract surgery:(2)

- Anterior capsule tear
- Posterior capsule tear with or without vitreous loss
- Choroidal effusion/ haemorrhage
- Zonular Dialysis
- Intra-operative pupil constriction needing intervention
- Dropped lens fragments or nucleus

Expected trial complications specifically for the **laser assisted** arm:

- Failure to dock to laser
- Aborted or incomplete laser delivery
- Incomplete capsulotomy identified in surgery, requiring manual completion
- Laser delivery to Inappropriate structure of eye

Expected post-operative complications for **both** arms:

- Post-operative uveitis
- Endophthalmitis
- Macular oedema
- Retinal tear or retinal detachment
- Elevated intraocular pressure requiring treatment
- Medication allergy or intolerance
- Corneal oedema
- Vitreous to wound
- Other ocular surgery

#### 5.11.3.6 Notifications

##### 5.11.3.6.1 Notifications by the Investigator to CCTU

CCTU must be notified of all SAEs immediately but not later than 24 hours of the investigator becoming aware of the event.

Investigators should notify CCTU of any SAEs and other Notifiable Adverse Events (NAEs) occurring from the time of randomisation until 12 months following surgery for all participants.

The SAE form must be completed by the investigator (the consultant named on the delegation of responsibilities list who is responsible for the participant’s care) or clinical delegate, with attention paid to the grading, causality and expectedness of the event. In the absence of the responsible investigator, the SAE form should be completed and signed by a member of the site trial team and emailed as appropriate within the timeline. The responsible investigator should check the SAE form at the earliest opportunity, make any changes necessary, sign and then email to CCTU. Detailed written reports should be completed as appropriate. Systems will be in place at the site to enable the investigator to check the form for clinical accuracy as soon as possible.

The minimum criteria required for reporting an SAE are the trial number and date of birth, name of reporting investigator and sufficient information on the event to confirm seriousness. Any further information regarding the event that is unavailable at the time of the first report should be sent as soon as it becomes available.

The SAE form and any follow-up forms must be scanned and sent to UCL CCTU in an encrypted format. Details of the method of transfer will be given at the Site Initiation Visit and documented in the Safety Management Plan. Participants must be followed up until clinical recovery is complete and laboratory results have returned to normal or baseline values, or until the event has stabilised. Follow-up should continue after completion of protocol treatment and/or trial follow-up if necessary. Follow-up SAE forms (clearly marked as follow-up) should be completed and sent to CCTU as further information becomes available. Additional information and/or copies of test results etc may be provided separately. The participant must be identified by trial number, date of birth and initials only. The participant’s name should not be used on any correspondence and should be blacked out and replaced with trial identifiers on any test results.

UCL CCTU must be notified of all Serious Unexpected Adverse Events within 24 hours of the investigator becoming aware of the event and must be notified to UCL CCTU until trial closure.

##### 5.11.3.6.2 CCTU responsibilities

The Chief Investigator (CI or a medically qualified delegate) will review all SAE reports received. In the event of disagreement between the causality assessment given by the local investigator and the CI, both opinions and any justifications will be provided in subsequent reports.

The delegated staff at CCTU will review the assessment for expectedness and, after discussion with the CI, may over-rule the local investigator assessment of expectedness for the purposes of onward reporting.

CCTU is undertaking the duties of trial sponsor and is responsible for the reporting of related unexpected SAEs to the REC as appropriate. Individual SAEs that are related and unexpected must be reported to the REC within 15 days of the Chief Investigator becoming aware of the event. Fatal and life threatening Serious Unexpected Adverse Events must be reported to the REC within 7 days of UCL CCTU becoming aware of the event; other Serious Unexpected Adverse Events must be reported within 15 days.

CCTU will keep investigators informed of any safety issues that arise during the course of the trial.

### 5.11.4 Quality Assurance and Control

#### 5.11.4.1 Risk Assessment

The Quality Assurance (QA) and Quality Control (QC) considerations for the **FACT** trial are based on the standard CCTU Quality Management Policy that includes a formal Risk Assessment, and that acknowledges the risks associated with the conduct of the trial and proposals of how to mitigate them through appropriate QA and QC processes. Risks are defined in terms of their impact on: the rights and safety of participants; project concept including trial design, reliability of results and institutional risk; project management; and other considerations.

QA is defined as all planned and systematic actions established to ensure the trial is performed and data generated, documented and/or recorded and reported in compliance with the principles of GCP and applicable regulatory requirements. QC is defined as the operational techniques and activities performed within the QA system to verify that the requirements for quality of the trial related activities are fulfilled.

#### 5.11.4.2 Central Monitoring at CCTU

CCTU staff will review Case Report Form (CRF) data for errors and missing key data points. The trial database will also be programmed to generate reports on errors and error rates. Essential trial issues, events and outputs, including defined key data points, will be detailed in the **FACT** trial Data Management Plan.

#### 5.11.4.3 On-site Monitoring

The frequency, type and intensity of routine and triggered on-site monitoring will be detailed in the **FACT** Quality Management and Monitoring Plan (QMMP). The QMMP will also detail the procedures for review and sign-off of monitoring reports. In the event of a request for a trial site inspection by any regulatory authority UCL CCTU must be notified as soon as possible.

##### 5.11.4.3.1 Direct access to participant records

Participating investigators must agree to allow trial related monitoring, audits, REC review and regulatory inspections, by providing access to source data and other trial related documentation as required. Participant consent for this must be obtained as part of the informed consent process for the trial.

#### 5.11.4.4 Trial Oversight

Trial oversight is intended to preserve the integrity of the trial by independently verifying a variety of processes and prompting corrective action where necessary. The processes reviewed relate to participant enrolment, consent, eligibility, and allocation to trial groups; adherence to trial interventions and policies to protect participants, including reporting of harms; completeness, accuracy and timeliness of data collection; and will verify adherence to applicable policies detailed in the Compliance section of the protocol. Independent trial oversight complies with the CCTU trial oversight policy.

In multi-centre trials this oversight is considered and described both overall and for each recruiting centre by exploring the trial dataset or performing site visits as described in the **FACT** Quality Management and Monitoring Plan.

##### 5.11.4.4.1 Trial Management Team

The Trial Management Team (TMT) will be set up to assist with developing the design, co-ordination and day to day operational issues in the management of the trial, including budget management.

##### 5.11.4.4.2 Trial Management Group

A Trial Management Group (TMG) will be set up to assist with developing the design, co-ordination and strategic management of the trial. The membership, frequency of meetings, activity (including trial conduct and data review) and authority will be covered in the TMG terms of reference.

##### 5.11.4.4.3 Independent Trial Steering Committee

The Independent Trial Steering Committee (TSC) is the independent group responsible for oversight of the trial in order to safeguard the interests of trial participants. The TSC provides advice to the CI, CCTU, the funder and sponsor on all aspects of the trial through its independent Chair. The membership, frequency of meetings, activity (including trial conduct and data review) and authority will be covered in the UCL CCTU TSC terms of reference.

##### 5.11.4.4.4 Independent Data Monitoring Committee

The Independent Data Monitoring Committee (IDMC) is the only oversight body that has access to unmasked accumulating comparative data. The IDMC is responsible for safeguarding the interests of trial participants, monitoring the accumulating data and making recommendations to the TSC on whether the trial should continue as planned. The membership, frequency of meetings, activity (including review of trial conduct and data) and authority will be covered in the UCL CCTU IDMC terms of reference. The IDMC will consider data in accordance with the statistical analysis plan and will advise the TSC through its Chair.

##### 5.11.4.4.5 Trial Sponsor

The role of the sponsor is to take on responsibility for securing the arrangements to initiate, manage and finance the trial. UCL is the trial sponsor and has delegated the duties as sponsor to CCTU via a signed letter of delegation.

# 6 Ethics and Dissemination

## 6.1 Research Ethics Approval

Before initiation of the trial at any clinical site, the protocol, all informed consent forms and any material to be given to the prospective participant will be submitted to the relevant REC for approval. Any subsequent amendments to these documents will be submitted for further approval. Before initiation of the trial at each additional clinical site, the same/amended documents will be submitted for local Research and Development (R&D) approval.

The rights of the participant to refuse to participate in the trial without giving a reason must be respected. After the participant has entered the trial, the clinician remains free to give alternative treatment to that specified in the protocol, at any stage, if s/he feels it to be in the best interest of the participant. The reasons for doing so must be recorded. After randomisation the participant must remain within the trial for the purpose of follow up and data analysis according to the treatment option to which they have been allocated. However, the participant remains free to change their mind at any time about the protocol treatment and follow-up without giving a reason and without prejudicing their further treatment.

## 6.2 Competent Authority Approvals

This is not a Clinical Trial of an Investigational Medicinal Product (IMP) as defined by the EU Directive 2001/20/EC. Therefore, a CTA is not required in the UK.

The progress of the trial, safety issues and reports, including expedited reporting of unexpected SAEs, will be reported to the REC in accordance with relevant national and local requirements and practices.

## 6.3 Other Approvals

The protocol will be submitted by those delegated to do so to the relevant R&D department of each participating site. A copy of the local R&D approval (or other relevant approval as above) and of the Participant Information Sheet (PIS) and consent form on local headed paper must be forwarded to CCTU before participants are randomised to the trial.

The protocol has received formal approval and methodological, statistical, clinical and operational input from the CCTU Protocol Review Committee.

## 6.4 Protocol Amendments

The CCTU will ensure that the trial protocol, patient information sheet, consent form, GP letter and submitted supporting documents have been approved by the research ethics committee and site Research & Development department prior to any patient recruitment. The protocol and all agreed substantial protocol amendments, will be documented and submitted for ethical approval prior to implementation.

## 6.5 Consent or Assent

Patients will be provided with a Patient Information Sheet (PIS) and given time to read it fully. Following a discussion with a medical qualified investigator or suitable trained and authorised delegate, any questions will be satisfactorily answered and if the participant is willing to participate, written informed consent will be obtained. During the consent process it will be made completely and unambiguously clear that the participant is free to refuse to participate in all or any aspect of the trial, at any time and for any reason, without incurring any penalty or affecting their treatment.

Consent will be re-sought if new information becomes available that affects the participant’s consent in any way. This will be documented in a revision to the patient information sheet and the participant will be asked to sign an updated consent form. These will be approved by the ethics committee prior to their use.

A copy of the approved consent form is available from the CCTU trial team.

## 6.6 Confidentiality

Data protection and information governance principles will be followed throughout the study, which will be overseen by the Trial Manager and Clinical Project Manager based at CCTU. Any confidentiality concerns expressed by potential patients will be addressed prior to providing informed consent.

Each patient will be assigned a unique trial participant identification number at randomisation. This number will be used on all trial-related documentation in place of personal identifiable data and used to identify patients on the CRFs. Patient identifiable information will be held securely at the sites and will be removed from documents and replaced with the trial number in the event of being sent off-site. Patient names will not be passed to anyone outside the research team who is not involved in the trial.

The records obtained during the trial, as well as related health records, will remain strictly confidential at all times. The information will be held securely on paper and electronically at the treating hospital under the provisions of the 1998 Data Protection Act. Information will be transferred from hospital sites to UCL CCTU on CRFs to enable analysis of the trial results to be undertaken. Patient names will only appear on their consent form, which will be kept at the hospital site in the medical notes, a copy will not be sent to the CCTU.

Patient records will be available to people authorised to work on the trial within NHS Trusts but may also need to be made available to people authorised by the Sponsor for monitoring and audit purposes. By signing the consent form patients agree to this access for the FACT trial and any further research that may be conducted in relation to it, even if they withdraw from the trial. When a patient withdraws consent from the trial, unless they object, their data will remain on file and will be included in the final trial analysis.

All trial staff will have a duty of confidentiality to participants in the FACT trial.

## 6.7 Declaration of Interests

The investigators named on the protocol have no financial or other competing interests that impact on their responsibilities towards the scientific value or potential publishing activities associated with the trial.

## 6.8 Indemnity

UCL holds insurance to cover participants for injury caused by their participation in the clinical trial. Participants may be able to claim compensation if they can prove that UCL has been negligent. However, as this clinical trial is being carried out in a hospital, the hospital continues to have a duty of care to the participant in the clinical trial. UCL does not accept liability for any breach in the hospital’s duty of care, or any negligence on the part of hospital employees. This applies whether the hospital is an NHS Trust or not. This does not affect the participant’s right to seek compensation via the non-negligence route.

Participants may also be able to claim compensation for injury caused by participation in this clinical trial without the need to prove negligence on the part of UCL or another party. Participants who sustain injury and wish to make a claim for compensation should do so in writing in the first instance to the Chief Investigator, who will pass the claim to UCL’s insurers, via the Sponsor’s office.

Hospitals selected to participate in this clinical trial shall provide clinical negligence insurance cover for harm caused by their employees and a copy of the relevant insurance policy or summary shall be provided to UCL, upon request.

## 6.9 Finance

The **FACT** trial is fully funded by a National Institute of Health Research Health Technology Assessment grant number 13/04/46. It is not expected that any further external funding will be sought.

## 6.10 Archiving

The investigators agree to archive and/or arrange for secure storage of **FACT** trial materials and records for a minimum of 5 years after the close of the trial unless otherwise advised by the CCTU.

## 6.11 Access to Data

Requests for access to trial data will be considered, and approved in writing where appropriate, after formal application to the TMG/TSC. Considerations for approving access are documented in the TMG/TSC Terms of Reference.

## 6.12 Ancillary and Post-trial Care

Once the trial has come to an end any further treatment to trial participants will be provided as per the standard of care at the local sites.

## 6.13 Publication Policy

### 6.13.1 Trial Results

The trial will be performed and reported in accordance with the CONSORT guidance.

The results of the trial will be disseminated regardless of the direction of effect.

Clinical and patient reported outcomes will be reported at 3 months after the last participant recruited (timing of the primary outcome). Participants are followed to 12 months post randomisation. The health economic analysis and 12 month follow up will be reported in subsequent reports. The overall HTA monograph will be reported after the last participants has reached the 12 month milestone.

Trial findings will be disseminated to all potential beneficiaries of the research including patients, carers and relatives, and also doctors, advisory bodies and health care Commissioners. This will take the form of papers in high impact open access (included in the budget) medical journals and also presentations at national and international medical conferences. We will seek publication of the trial protocol once finalised. Trial results will also be disseminated to the trial patients in a one-page summary written in lay language.

The trial will inform NHS service providers, commissioners, patients and the clinical community about the effectiveness of these two methods of cataract surgery.

### 6.13.2 Authorship

Publications generated from the trial will be attributed to the FACT Trial Management Group, which will consist of all those who have wholeheartedly collaborated in the trial. The main report will be drafted by the TMG, and the final version will be reviewed by the TSC before submission for publication. TMG members will be named and their affiliations listed in the main report. All publications will be in compliance with the CCTU Publication Policy.

# 7 Ancillary Studies

The FACT trial does not have any ancillary studies.

# 8 Protocol Amendments

Version 1.0 – updated to address TSC responses to follow up timelines and eligibility criteria, updates also made to safety reporting and to add clarifications to various sections.

Version 3.0 – updated to address TMG responses to further clarification on the masking procedures and the IDMC responses to safety reporting, adding clarifications to various sections.

The title of the trial has been updated in accordance to the CONSORT guidelines.

Version 4.0 – updated to address handling of missing data in the statistical analysis plan and to clarify onward reporting of related and unexpected Serious Adverse Events (SAEs). An update to the dissemination of the trial results has also been addressed and clarified.

# 9 References

1. Evans JR, Fletcher AE, Wormald RPL, MRC Trial of Assessment and Management of Older People in the Community. Causes of visual impairment in people aged 75 years and older in Britain: an add-on study to the MRC Trial of Assessment and Management of Older People in the Community. Br J Ophthalmol. 2004 Mar;88(3):365–70.

2. Jaycock P, Johnston RL, Taylor H, Adams M, Tole DM, Galloway P, et al. The Cataract National Dataset electronic multi-centre audit of 55 567 operations: updating benchmark standards of care in the United Kingdom and internationally. Eye. 2007;23(1):38–49.

3. NICE, National Institute for Health and Care Excellence. Guide to the methods of technology appraisal 2013: Process and Methods Guide. [Internet]. 2013 [cited 2014 Jan 23]. Available from: http://publications.nice.org.uk/guide-to-the-methods-of-technology-appraisal-2013-pmg9

4. NOD. Royal College of Ophthalmologists National Ophthalmic Database [Internet]. 2013. Available from: http://www.rcophth.ac.uk/page.asp?section=668&sectionTitle=National+Ophthalmology+Database

5. Johansson B, Lundström M, Montan P, Stenevi U, Behndig A. Capsule complication during cataract surgery: Long-term outcomes: Swedish Capsule Rupture Study Group report 3. J Cataract Refract Surg. 2009;35(10):1694–8.

6. Qatarneh D, Mathew RG, Palmer S, Bunce C, Tuft S. The economic cost of posterior capsule tear at cataract surgery. Br J Ophthalmol. 2012;96(1):114–7.

7. Jakobsson G, Montan P, Zetterberg M, Stenevi U, Behndig A, Lundström M. Capsule complication during cataract surgery: Retinal detachment after cataract surgery with capsule complication: Swedish Capsule Rupture Study Group report 4. J Cataract Refract Surg. 2009;35(10):1699–705.

8. Narendran N, Jaycock P, Johnston RL, Taylor H, Adams M, Tole DM, et al. The Cataract National Dataset electronic multicentre audit of 55,567 operations: risk stratification for posterior capsule rupture and vitreous loss. Eye. 2009;23(1):31–7.

9. Friedman NJ, Palanker DV, Schuele G, Andersen D, Marcellino G, Seibel BS, et al. Femtosecond laser capsulotomy. J Cataract Refract Surg. 2011 Jul;37(7):1189–98.

10. Nagy ZZ, Kránitz K, Takacs AI, Miháltz K, Kovács I, Knorz MC. Comparison of intraocular lens decentration parametres after femtosecond and manual capsulotomies. J Refract Surg. 2011 Aug;27(8):564–9.

11. Kránitz K, Takacs A, Miháltz K, Kovács I, Knorz MC, Nagy ZZ. Femtosecond laser capsulotomy and manual continuous curvilinear capsulorrhexis parametres and their effects on intraocular lens centration. J Refract Surg Thorofare NJ 1995. 2011 Aug;27(8):558–63.

12. Kránitz K, Miháltz K, Sándor GL, Takacs A, Knorz MC, Nagy ZZ. Intraocular lens tilt and decentration measured by Scheimpflug camera following manual or femtosecond laser-created continuous circular capsulotomy. J Refract Surg. 2012 Apr;28(4):259–63.

13. Miháltz K, Knorz MC, Alió JL, Takács AI, Kránitz K, Kovács I, et al. Internal aberrations and optical quality after femtosecond laser anterior capsulotomy in cataract surgery. J Refract Surg. 2011 Oct;27(10):711–6.

14. Abell RG, Kerr NM, Vote BJ. Toward zero effective phacoemulsification time using femtosecond laser pretreatment. Ophthalmology. 2013 May;120(5):942–8.

15. Abell RG, Kerr NM, Vote BJ. Femtosecond laser-assisted cataract surgery compared with conventional cataract surgery. Clin Experiment Ophthalmol. 2013 Jul;41(5):455–62.

16. Conrad-Hengerer I, Hengerer FH, Schultz T, Dick HB. Effect of femtosecond laser fragmentation on effective phacoemulsification time in cataract surgery. J Refract Surg Thorofare NJ 1995. 2012 Dec;28(12):879–83.

17. Conrad-Hengerer I, Hengerer FH, Schultz T, Dick HB. Effect of femtosecond laser fragmentation of the nucleus with different softening grid sizes on effective phaco time in cataract surgery. J Cataract Refract Surg. 2012 Nov;38(11):1888–94.

18. Roberts TV, Lawless M, Bali SJ, Hodge C, Sutton G. Surgical outcomes and safety of femtosecond laser cataract surgery: a prospective study of 1500 consecutive cases. Ophthalmology. 2013 Feb;120(2):227–33.

19. Trikha S, Turnbull AMJ, Morris RJ, Anderson DF, Hossain P. The journey to femtosecond laser-assisted cataract surgery: new beginnings or a false dawn? Eye. 2013 Apr;27(4):461–73.

20. NIHR Horizon Scanning Centre. Femtosecond lasers for cataract surgery [Internet]. 2012 [cited 2013 Nov 20]. Available from: http://www.hsc.nihr.ac.uk/topics/femtosecond-lasers-for-cataract-surgery/

21. Day A, Gore D, Bunce C. Laser assisted versus manual phacoemulsification for lens extraction (Protocol). Protoc Cochrane Database Syst Rev. 2013;(9).

22. Conrad-Hengerer I, Al Juburi M, Schultz T, Hengerer FH, Dick HB. Corneal endothelial cell loss and corneal thickness in conventional compared with femtosecond laser-assisted cataract surgery: three-month follow-up. J Cataract Refract Surg. 2013 Sep;39(9):1307–13.

23. Reddy KP, Kandulla J, Auffarth GU. Effectiveness and safety of femtosecond laser-assisted lens fragmentation and anterior capsulotomy versus the manual technique in cataract surgery. J Cataract Refract Surg. 2013 Sep;39(9):1297–306.

24. FEMCAT. Economic Evaluation of Femtosecond Laser Assisted Cataract Surgery (FEMCAT) [Internet]. [cited 2014 Jan 1]. Available from: http://clinicaltrials.gov/ct2/show/NCT01982006

25. Barkun JS, Aronson JK, Feldman LS, Maddern GJ, Strasberg SM, Balliol Collaboration, et al. Evaluation and stages of surgical innovations. Lancet. 2009 Sep 26;374(9695):1089–96.

26. Lundström M, Pesudovs K. Catquest-9SF patient outcomes questionnaire: nine-item short-form Rasch-scaled revision of the Catquest questionnaire. J Cataract Refract Surg. 2009 Mar;35(3):504–13.

27. Lundström M, Albrecht S, Håkansson I, Lorefors R, Ohlsson S, Polland W, et al. NIKE: a new clinical tool for establishing levels of indications for cataract surgery. Acta Ophthalmol Scand. 2006;84(4):495–501.

28. Patel A, Rendu A, Moran P, Leese M, Mann A, Knapp M. A comparison of two methods of collecting economic data in primary care. Fam Pr. 2005;22:323–7.

29. Longworth L, Yang Y, Young T, Mulhern B, Hernández Alava M, Mukuria C, et al. Use of generic and condition-specific measures of health-related quality of life in NICE decision-making: a systematic review, statistical modelling and survey. Health Technol Assess Winch Engl. 2014 Feb;18(9):1–224.

30. O’Hagan A, Stevens JW. A framework for cost-effectiveness analysis from clinical trial data. Health Econ. 2001 Jun;10(4):303–15.

31. O’Hagan A, Stevens J, Montmartin J. Bayesian cost-effectiveness analysis from clinical trial data. Stat Med. 2001;20:733–53.

32. Chan AW, Tetzlaff JM, Altman DG et Al. SPIRIT 2013 Statement: Defining Protocol Items for Clinical Trials. *Ann Intern Med* 2013; 158:200-207.

33. Chan AW, Tetzlaff, Gotzsche et Al. SPIRIT 2013 explanation and elaboration: guidance for protocols of clinical trials. *BMJ* 2013; 346: e7586.

#

# 10 Appendices

## 10.1 Specular microscopy for obtaining central corneal endothelial cell density – Standard Operating Procedure

- Central corneal endothelial cell density (CCED) and morphology will be measured using a non-contact specular microscope.
- Coefficient of variation of cell size (COV), and the percentage of hexagonality (HEX) will also be recorded to indicate the degree of polymorphism and pleomorphism respectively.
- Calculation of endothelial cell loss:
- Percentage change in CCED at each time point from baseline

= (Preop CCED – CCED at each time point)/Preop CCED x 100

1. Visual acuity (logMAR) with the patient’s usual method of correction (current glasses or unaided). [↑](#footnote-ref-2)
